# Embodied postural dynamics underlie behavioral diversity in a benthic sessile chordate

**DOI:** 10.1016/j.isci.2026.116104

**Published:** 2026-05-26

**Authors:** Oleg Tolstenkov, Sissel Norland, Marios Chatzigeorgiou

**Affiliations:** 1 Michael Sars Centre, Faculty of Science and Technology, University of Bergen, 5006 Bergen, Norway; 2 Department of Life Sciences, Imperial College, London SW7 2AZ, UK

**Keywords:** biological sciences, zoology, evolutionary biology, biomechanics

## Abstract

Sessile benthic animals are thought to have limited behavioral repertoires due to immobility and simple nervous systems. Here, we characterize the spontaneous and stimulus-evoked behaviors of adult *Ciona intestinalis*, a basal chordate with a sessile adult stage. Using time-lapse imaging and markerless pose estimation, we extracted detailed body outlines and kinematic features and showed that *Ciona* organizes behavior into three postural engagement states distinguished by configuration, kinematics, temporal structure, and transitions. These states are strongly modulated by external cues: mechanical pokes across the body and water flow elicit distinct sensorimotor responses, while chemical cues drive compound-specific motor patterns. Dimensionality reduction revealed that five “eigencionas’’ capture most postural variance, and spatiotemporal embedding uncovered 16 stereotyped behavioral modules deployed in a context-dependent manner. Hidden Markov modeling showed that mechanical, hydrodynamic, and chemical stimuli reorganize transitions within a shared state space. Together, these results reveal a rich, modular behavioral repertoire in adult *Ciona*.

## Highlights

• Sessile adult *Ciona* displays a rich repertoire of spontaneous and evoked behaviors
• Behavior is organized into discrete postural states with stereotyped transitions
• Mechanical, hydrodynamic, and chemical cues elicit stimulus-specific behaviors
• Sensory cues dynamically reshape postural states and behavioral transitions

## Graphical abstract

**Figure:**
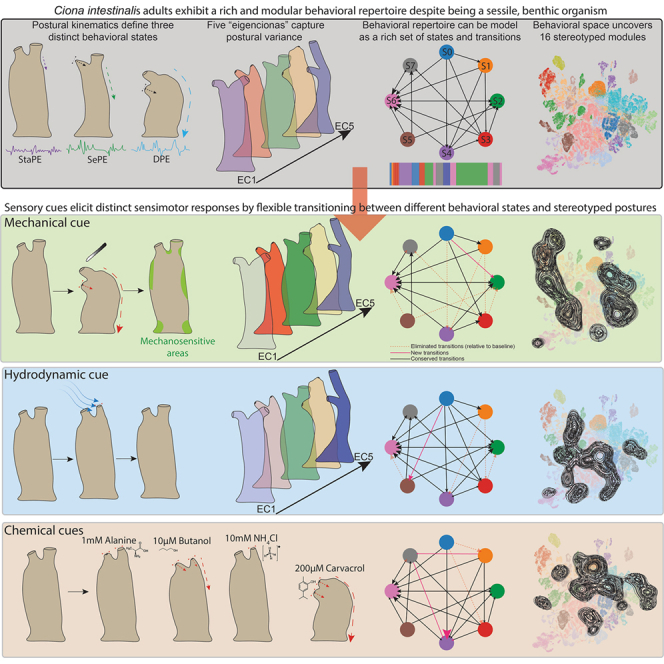

## Introduction

The fossil record suggests that a significant fraction of the Ediacaran biota (575-542 Mya) was composed of benthic marine organisms with a tubular, oval, branched, or petal-shaped form.^1^^,^^2^^,^^3^ A lot of these were sessile, anchored to the benthos.^4^ They would acquire their food either via osmotrophy or suspension feeding,^2^^,^^5^ and some would show a very limited behavioral repertoire (e.g., aligning with the current flow).^6^

The Ediacaran biota abruptly disappeared 542 Mya, likely because of mass extinction and/or through biological interactions with the animals of the Cambrian explosion. Rapidly evolving animals in the Cambrian explosion included epibenthic sessile species capable of predation or filter-feeding, such as stem-group ctenophores, cnidarians, sponges, brachiopods, and tunicates.^7^^,^^8^^,^^9^^,^^10^^,^^11^^,^^12^^,^^13^^,^^14^

Numerous sessile benthic organisms exist among extant metazoans. The majority arise from a pelagobenthic cycle, where a planktonic larva undergoes the transformative process of metamorphosis to become a sexually mature sessile, epibenthic adult. A biphasic life cycle is often associated with dramatic morphological, physiological, and behavioral changes between larval and adult stages.^15^^,^^16^ These changes can be beneficial in several ways, including occupying distinct ecological niches and specializing in different activities. For example, the freely swimming larvae can take advantage of marine currents to travel far away from the site of release,^17^ while the adults can be benthic, sessile filter feeders, which grow and spawn a large number of eggs and sperm. Larval behaviors and neurophysiology are being extensively studied across a broad diversity of marine species with a biphasic life cycle.^18^^,^^19^^,^^20^^,^^21^^,^^22^^,^^23^^,^^24^^,^^25^^,^^26^^,^^27^^,^^28^^,^^29^ However, we have a much more fragmentary understanding of the adult behavioral repertoire and sensory physiology, since studies of the benthic (and often sessile) adult stage are sparse.^30^^,^^31^^,^^32^

Ascidians are marine invertebrates with a biphasic life cycle. As the sister group to vertebrates, they have become important models for studying chordate development, physiology, behavior, and ecology.^33^ Solitary ascidians such as the emerging model organism *Ciona intestinalis*, have a freely swimming lecithotrophic larva, which eventually settles and metamorphoses to a benthic, sessile, and filter-feeding adult. The central nervous system of *Ciona* larvae shares key characteristics with other chordates, and thus it is the focus of intense research across scales from molecules to behaviors.^18^^,^^19^^,^^29^^,^^34^^,^^35^^,^^36^^,^^37^^,^^38^^,^^39^^,^^40^^,^^41^^,^^42^^,^^43^^,^^44^^,^^45^ During the process of metamorphosis, most larval structures degenerate, and they are replaced by adult tissues.^46^^,^^47^^,^^48^ These adult structures originate primarily from various pools of undifferentiated progenitor cells (rudiments) present in the larvae.^48^^,^^49^^,^^50^^,^^51^^,^^52^ Despite being an order of magnitude larger in terms of number of neurons than the larval nervous system, the adult brain of *Ciona* is considered a relatively uninteresting organ, potentially due to a misconception that a sessile organism does not need a brain since it is not motile. Adult ascidians' brains are adapted to sessility, evolving sensory organs and reflexes suitable to their benthic environmental niche.^43^^,^^53^^,^^54^^,^^55^^,^^56^^,^^57^ The nervous system of the adult *Ciona* is composed of the cerebral ganglion and the neural gland, which make up the CNS and the dorsal (D) strand plexus, the visceral nerves, and a more diffuse network of anterior and posterior nerves that make up the peripheral nervous system.^56^^,^^57^^,^^58^^,^^59^^,^^60^

To date, a small number of neurophysiological studies have been performed in adult ascidians. Using primarily electrophysiological assays, they have shown that ascidian adults can detect and respond to stimuli such as light, water flow, acute mechanical poke, vibrations of different frequencies, and they can distinguish between particles of different sizes that pass by their siphons.^56^^,^^57^ However, this physiological data is still missing behavioral correlates. Historically, animal behavior tracking tools required manual annotation of numerous video frames, and early tracking software focused either on following the center of mass of freely moving animals without providing postural information or extracting the midline and/or contour of a few predefined body shapes.^61^^,^^62^^,^^63^ The advent of convolutional neural networks and markerless pose tools for tracking a huge diversity of animals, such as DeepLabCut,^64^^,^^65^ SLEAP,^66^ and others^67^^,^^68^ offers an unprecedented opportunity for the automatic extraction of the ascidian adult body’s outline, capturing most of the pose information collected in video recordings.

Here, we leverage time-lapse video recordings of *Ciona* adults in combination with the markerless pose estimation tool DeepLabCut^64^ to map the *Ciona intestinalis* adult behavioral repertoire. We obtained a detailed overview of shape-based features and body part kinematics. Using these shape features and body part kinematics, we find that *Ciona* adults spend most of their time in one of the three major behavioral states. These states differ from each other in terms of postural features and kinematics, duration, and frequency of occurrence. Using dimensionality reduction, we derived lower-dimensional representations of body postures, which we define as “Eigencionas” (ECs). With these ECs, we can explain the majority of postural variance in the *Ciona* adults.

In addition, we exposed the animals to water flow, applied mechanical poke stimuli across seven distinct locations on the *Ciona* adult body, and delivered four different chemical stimuli to study behavioral responses to qualitatively different sensory stimuli. Each of these stimulus modalities elicited distinct sensory responses, as quantified by postural features/kinematics, spatiotemporal embedding, and Hidden Markov modeling (HMM).

## Results

### Parametrization of adult *Ciona* using interpretable features reveals three behavioral states

**Figure 1:**
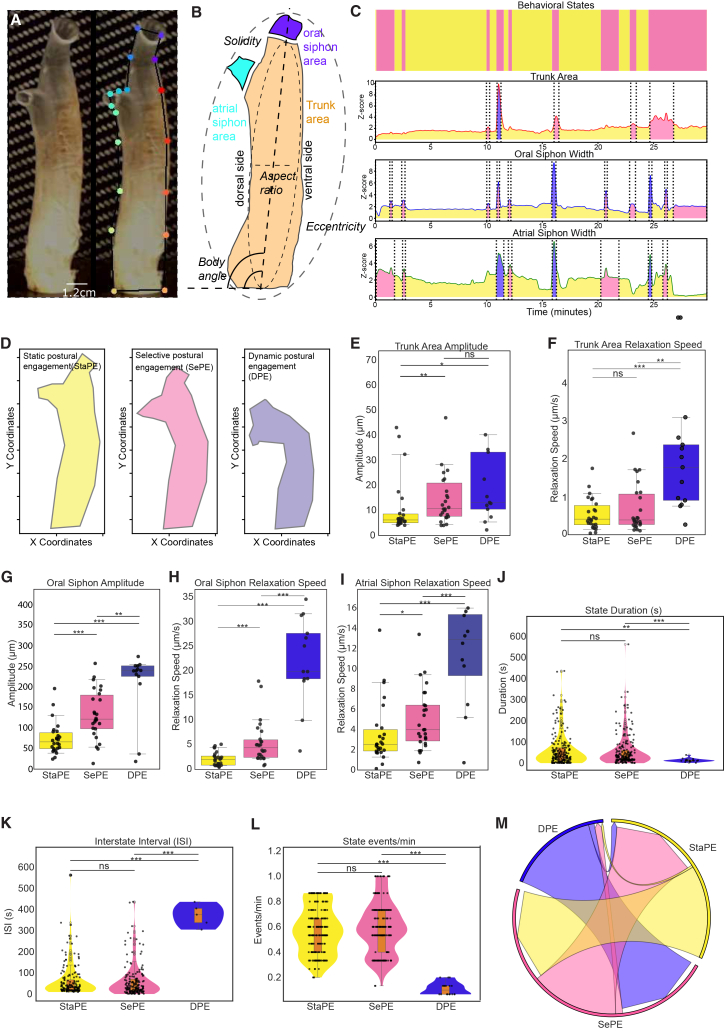
Parametrization of adult *Ciona* using interpretable features reveals three behavioral states (A) Video frame of a Ciona intestinalis adult without (left) and with (right) DeepLabCut-based annotation of key contour landmarks (see also Video S1). Scale bars, 1.2 cm. (B) Schematic of adult *Ciona* body parts and morphological features used in our analysis. (C) Ethogram (top panel) of a single *Ciona* adult video-recorded for 30 min exhibiting spontaneous behaviors. Coloring corresponds to the behavioral modules defined based on threshold values of trunk area, oral siphon width, and atrial siphon width. The corresponding z-scored time series of these features for this individual *Ciona* are shown in the lower three panels. (D) Reconstruction of *Ciona* contours from individual video frames that have been classified as belonging to one of the three states: dynamic postural engagement (DPE), selective postural engagement (SePE), and static postural engagement (StaPE) (Please see Table S1 for an extended description of each state). (E–I) Boxplots quantify different postural features and body part kinematics were used in the characterization of the three behavioral states. Mann-Whitney U tests were used for statistical analysis (ns *p* > 0.05; ∗*p* < 0.05; ∗∗*p* < 0.005; and ∗∗∗*p* < 0.0001). Number of animals used *n* = 43. (J–L) Violin plots quantify the temporal features in each of the three behavioral states. (M) Chord diagram shows the transitions between the three behavioral states in spontaneously behaving animals. For panels E-L plots show median with interquartile range (IQR: 25th–75th percentile) and whiskers extend to 1.5× the IQR.

Using an inexpensive camera setup, we recorded high-resolution movie data of adult *Ciona intestinalis* individuals performing spontaneous and stimulus-evoked behaviors. To automatically extract *Ciona* adult posture, we trained a DeepLabCut model, where we defined multiple points (18 points) along the animal’s body (Figure 1A; Video S1). By reconstructing the outline of the *Ciona* adult body, we could follow the opening and closing of the atrial and oral siphons, as well as changes in the posture of the trunk shape. Outline coordinates were converted to kinematic and postural metrics for the different animal body regions (Figure 1B). In addition, we extracted 30 elliptical Fourier descriptors (EFDs) to efficiently capture both gross and subtle changes in body posture over time (Figures S1A–S1C).

Video S1. Two adult Ciona intestinalis performing spontaneous behaviors Their outlines are marked with 18 key points by a DNN trained in DeepLabCut, related to Figure 1

Spontaneously behaving *Ciona* adults show variability in behavior across time (e.g., Video S1). In other animals, this variability is generated through rapid shifts in behavior as animals transition between different behavioral states.^69^^,^^70^ A plethora of behavioral states have been documented in the literature for animals that can freely locomote, however a lot of these states (e.g., roaming, dwelling, foraging, and escape) are not applicable to sessile organisms such as the benthic *Ciona* adult, which are fixed to one place permanently. To behave, sessile animals do not rely on locomotion, but rather they engage their body’s postural control system aimed at actively maintaining specific postures and transitioning between them. We define this process as postural engagement.

Analyzing the spontaneous behavior of adult *Ciona,* we identified three behavioral states, which we classified as dynamic postural engagement (DPE), selective postural engagement (SePE), and static postural engagement (StaPE) (Figures 1C, 1D, and S1D; Table S1). These states were defined by the synchronization of the contraction events in the three body regions characterized by the difference in various postural and kinematic parameters of the three body regions (Figures 1E–1I and S1E–S1P). In the case of the trunk, area amplitude and relaxation speed best differentiated the states (Figures 1E, 1F, and S1E). For the oral siphon region, aperture amplitude and relaxation speed were the most informative features (Figures 1G, 1H, and S1F–S1I). Finally, for the atrial siphon, relaxation speed was the most informative feature (Figures 1I and S1J–S1N). On occasion, we noticed that the adult *Ciona* bodies acquired a slightly bent posture, but neither body angle (the orientation of the main body axis relative to the horizontal) nor D/ventral (V) ratio metrics showed a significant difference (Figures S1O and S1P) between states during spontaneous behavior.

We next characterized the temporal features of the behavioral states. We found that the DPE state had the shortest duration (Figure 1J) and the lowest frequency of occurrence among the three states (Figures 1K and 1L). The temporal dynamics of the DPE and SePE states were not significantly different from each other (Figures 1J–1L). We also analyzed the transition probabilities between states. Animals in the intermediate SePE state could transition to either DPE or StaPE. However, we failed to identify direct transitions between the DPE and StaPE states. This is indicative of a stereotyped sequence of transitions in behavioral states in the absence of an external sensory stimulus (Figure 1M).

### Mechanical stimuli elicit distinct behavioral responses

**Figure 2:**
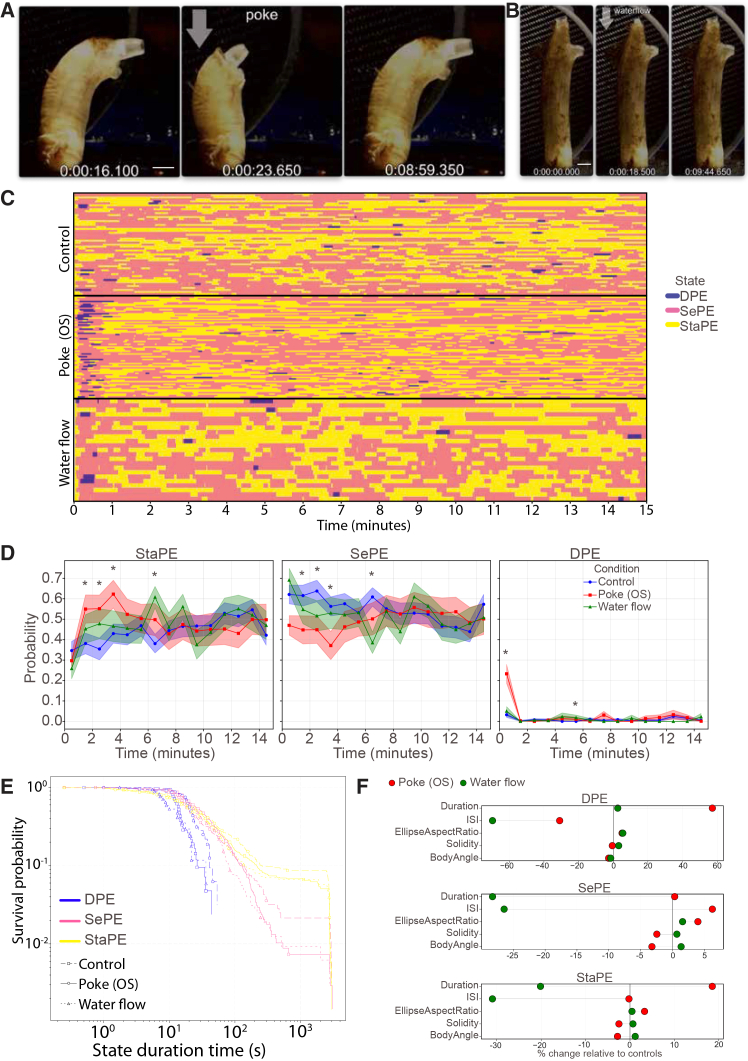
Mechanical stimuli elicit distinct behavioral responses (A and B) Video frames from two *Ciona* adults responding to a (A) mechanical poke and (B) water flow (see Videos S2 and S3). In both cases, the arrow indicates the time of stimulus application. Scale bars, 1.2 cm. (C) Ethograms of *Ciona* adults belonging to three groups (controls, i.e., no stimulus delivered, poke OS, or water flow). Blue color corresponds to dynamic postural engagement (DPE); pink corresponds to selective postural engagement (SePE), and yellow to static postural engagement (StaPE). (D) Line plots summarize the probability of an adult *Ciona* being in one of the three states during the video recordings as a function of the stimulus used (no stimulus/control) (number of animals = 52), poke OS (number of animals = 28), and water flow (number of animals = 23). We show the mean (thick lines) and the standard error of the mean) SEM shaded areas. For statistical analysis, we used the Kruskal-Wallis test (∗*p* < 0.05). (E) Quantification of the duration/persistence of each behavioral state as a function of stimulus used. (F) Plots quantify the percentage change relative to controls (no stimulus, baseline = 0) of various postural and temporal features across the three different behavioral states. Please see Table S2 for a summary description of body part movements in response to poke OS and water flow stimuli.

*Ciona* adults are equipped with mechanoreceptors on their siphons, and these are sensitive to water flow and poking, as demonstrated by electrophysiological studies.^56^^,^^57^ Here, we recorded and analyzed the behavioral responses of *Ciona* adults in response to mechanical poking and water flow (Figures 2A, 2B, and S2A–S2D; Tables S1, S2, S3; Videos S2 and S3). We found that a mechanical poke outside the oral siphon (poke OS) elicited a strong defensive response by entering a DPE state and performing a full contraction (Figures 2A and 2C, blue bars). A DPE state was observed with significantly higher probability immediately following a poke stimulus compared to water flow-stimulated and control unstimulated animals (Figure 2D). When mechanically poked animals exited the DPE state, they showed a higher probability of entering a StaPE (Figures 2C (pink bars) and 2D). This is in sharp contrast to what we observed in controls (no-stimulus), where this transition was never observed. This suggests that acute mechanical stimulation can introduce plasticity in the state transition dynamics.

Video S2. Adult *Ciona intestinalis* responding to a mechanical poke stimulus, related to Figure 2

Video S3. Adult *Ciona intestinalis* responding to a water flow stimulus, related to Figure 2

In contrast to a poke stimulus, a water flow stimulus did not induce an immediate transition to a DPE state, suggesting that it is perceived by the animal’s nervous system as a qualitatively different stimulus to poke (Figure 2D). However, an unexpected observation was that the probability of animals entering a DPE state (mostly in the form of full contraction events) was significantly higher at 5.5 min after water flow stimulation, suggesting that this stimulus may elicit a delayed behavioral response (Figure 2D).

Inspecting the ethograms of unstimulated and stimulated animals, we noticed that the duration of individual StaPE, SePE, and DPE events was different across the three treatments (Figure 2C). We found that in control unstimulated animals, the static state (StaPE) was the most long-lasting (Figure 2E, yellow line with squares), with SePE state events having shorter duration relative to StaPE events (Figure 2E, pink line with squares). The DPE state events were the most short-lived, which is reasonable given that this is an energetically costly state demanding the fast and recurrent contraction of multiple muscle sets along the animal’s body (Figure 2E, blue line with squares). We then asked whether mechanical stimulation in the form of poke or water flow altered the duration of individual events for each of the three states. The duration of individual events for StaPE and SePE was reduced in waterflow-stimulated animals compared to the unstimulated control experiments, but the duration of DPE events remained unaffected (Figures 2E and 2F). The duration of StaPE but not SePE events increased following a poke stimulus relative to controls (Figures 2E and 2F). In contrast, DPE events showed a significant increase in event duration in poked animals compared to unstimulated controls (Figures 2E and 2F). In addition, we quantified the interval between successive events of the same state (termed the interstate interval (ISI). A water flow stimulus resulted in a strong decrease in ISI for all three states compared to unstimulated controls (Figure 2F). Poke stimuli led to a significant reduction in ISI of DPE events (Figure 2F). Notably, when we compared transition probabilities between the three states in control, poke, and water flow-stimulated animals, we found that poke stimuli increased the transition probability from an SePE state to a DPE state, while water flow stimulation eliminated the transition from a DPE state to an StaPE state (Figures S2E and S2F). These surprising findings suggest that the nervous system of adult *Cionas* can modulate the duration and usage rate of these behavioral states in a sensory context-specific manner.

We then asked how poke and water flow stimuli altered the postural and kinematic features of the three regions (trunk, oral, and atrial siphons) across the three different behavioral states. Across all behavioral states, stimulus-evoked changes were most pronounced in the siphon features, followed by whole-body polygon metrics, whereas the trunk features exhibited relatively small deviations from baseline (Figures S3A–S3C).

Siphon kinematics displayed the largest and most stimulus-sensitive modulation. In the DPE state, poke stimuli induced substantial increases primarily in oral siphon area and width kinematics (Figure S3A). Water flow stimuli elicited strong changes in oral siphon width and trunk area relaxation speeds (Figure S3A). In the SePE state, atrial and oral siphon width relaxation speeds were strongly increased in poke-stimulated animals, while water flow stimuli induced limited changes to postural and kinematic features (Figure S3B). In the StaPE state, the biggest changes were observed in polygon area dynamics in response to poke stimuli (Figure S3C). Water flow stimuli led to modest increases in oral siphon width contraction and relaxation dynamics but very limited changes in trunk features (Figures S3A–S3C). Taken together, our data reveal a hierarchical organization of responsiveness and suggest that the siphon structures serve as the primary effectors of stimulus-driven movement, capable of rapid movement and large changes in aperture, while trunk and whole body (polygon) movements are relatively limited and constitute a secondary motor response to external perturbation.

### Five eigenciona shapes capture most of the postural variance of adult *Ciona*

**Figure 3:**
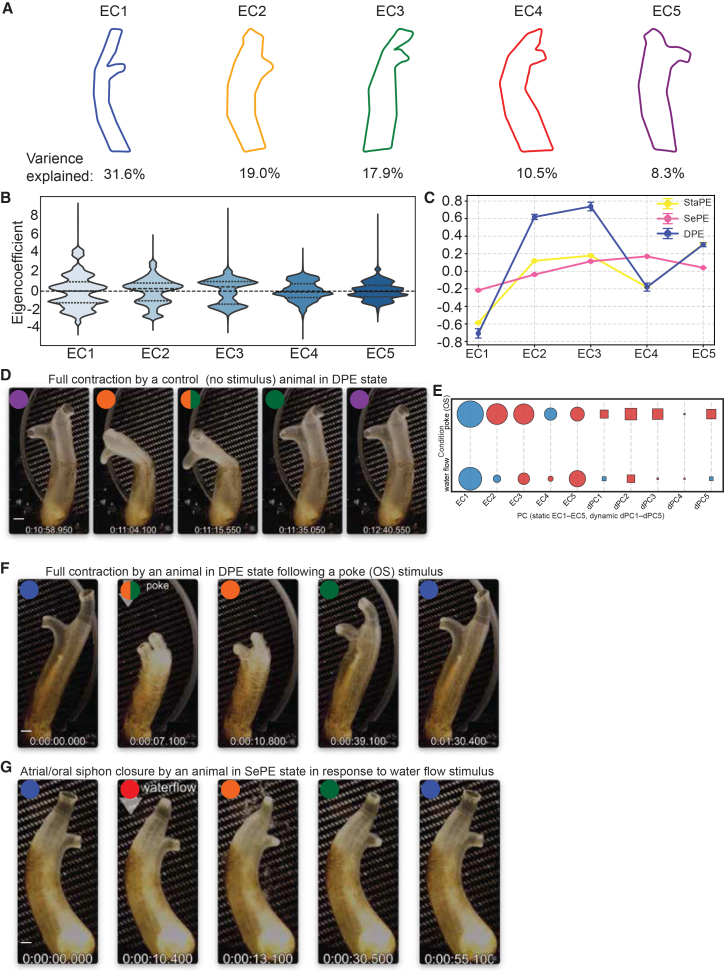
Five eigenciona shapes capture most of the postural variance of adult *Cion*a (A) Visualization of the top 5 eigencionas (PCs) obtained by an eigen decomposition of the covariance matrix, shown in descending order of the fraction of variance explained, (Please see Table S1 for an extended description of each eigenciona). (B) Distribution of the eigencoefficient values for all spontaneously behaving *Ciona* adults (*n* = 43) The outer filled shape represents Kernel density estimation (KDE). The thick dashed line of each plot corresponds to the median, and the thin dashed lines correspond to the IQR. (C) Mean eigenciona summarized by behavioral state. Circles indicate the mean value, and whiskers correspond to the S.E.M. (D) Video frames from a *Ciona* adult exhibiting a spontaneous full contraction as they enter a DPE state. Coloring of the circles and semi-circles indicates the eigencionas that capture the postures of the *Ciona* in the specific video frames (see Video S4). (E) Bubble grid chart showing the effects of oral siphon poking and water flow stimuli in the use of static (PCs) and dynamic (PCs derivatives) eigencionas. Circles refer to static EigenCionas and boxes refer to dynamic eigencionas. Color indicates an increase (Red) or decrease (Blue) in eigenciona use, with the radius indicating the mean value. Animals used for this analysis control = 52; poke OS = 28 and water flow = 23. (F and G) Video frames of individual *Ciona* showing a response to an OS poke stimulus (F)and to a water flow stimulus (G) (see Videos S5 and S6). Color(s) of the circles and semi-circles indicate the eigencionas that capture the postures of the Ciona in the specific video frames. Scale bars for panels D, F, and G correspond to 1.2 cm.

While biologically interpretable features such as amplitudes of trunk or siphon rim width, opening amplitude, aspect ratio, body angle, D/V ratio, and EFDs can describe postures with good accuracy, using these features comes at the price of very high dimensionality.^71^ We were thus interested in obtaining a simpler representation that described the range of postures that Ciona adults can attain as represented by the top ten harmonic (EFD) amplitudes without losing substantial information, since they explain over 95% of the total shape variance (Figures 3A and S4A).^72^^,^^73^ We performed principal component analysis (PCA) of these 10-dimensional vectors (amplitudes) and revealed that the first five components (hereafter described as eigencionas, EC1-EC5 or PC1-PC5) explained over 87% of the variance or dominant modes of body posture variation (Figures 3A and S4B). For any video frame, the body postures can be approximated as a linear combination of the five eigencionas: EC1 to EC5 (Figure 3A; Table S1). Therefore, the coefficients of the five eigencionas (or PC1-PC5) were leveraged for further analysis as a simplified but accurate description of body postures (Figure 3B). Interestingly, while postural quiescence and partial contraction states showed relatively balanced contributions across all ECs, DPE was dominated by EC2 and EC3, corresponding to postures with contracted body and closed siphons (Figures 3A, 3C, and 3D; Video S4). To further capture the temporal dynamics of shape transitions, we also computed frame-to-frame derivatives of the 10 EFD amplitudes and performed PCA on these dynamic features (Figures S3E and S3F). The resulting dynamic principal components or dPCs provided complementary information for state characterization, showing increasing dynamic contributions across most components from StaPE, SePE, and DPE states (Figures 3E and S4C–S4F).

Video S4. Adult *Ciona intestinalis* performing spontaneous behaviors, related to Figure 3

To define shape responses to mechanical (poke) and hydrodynamic (water flow) stimuli in an unsupervised manner, we analyzed both static (EC1:EC5) and dynamic (dEC1:dEC5) components of body shape variation based on EFD amplitudes and compared them to spontaneous behavior (Figure 3E). In both conditions, PC1 captured the dominant static shape variation. Under poke, additional static components (PC2 and PC3) contributed substantially, reflecting stimulus-driven shape modulation, consistent with previous analyses highlighting the role of EC2 and EC3 when animals are found in a DPE state. In contrast, under water flow, static variation beyond PC1 was reduced. Similarly, dynamic shape changes under poke were prominent, particularly in dEC2 and dEC3, indicating active modulation of body shape in response to the mechanical stimulus (Figures 3E and 3F; Videos S5 and S6). In water flow, dynamic components were largely suppressed, suggesting more stable postures with limited shape modulation (Figures 3E and 3G). These findings show that poke stimuli evoke both static and dynamic shape changes, while water flow induces primarily static adjustments with limited dynamics.

Video S5. Adult *Ciona intestinalis* responding to a mechanical poke stimulus, related to Figure 3

Video S6. Adult *Ciona intestinalis* responding to a water flow stimulus, related to Figure 3

### Regional dependence of behavioral responses to mechanical poke stimuli in adult *Ciona*

**Figure 4:**
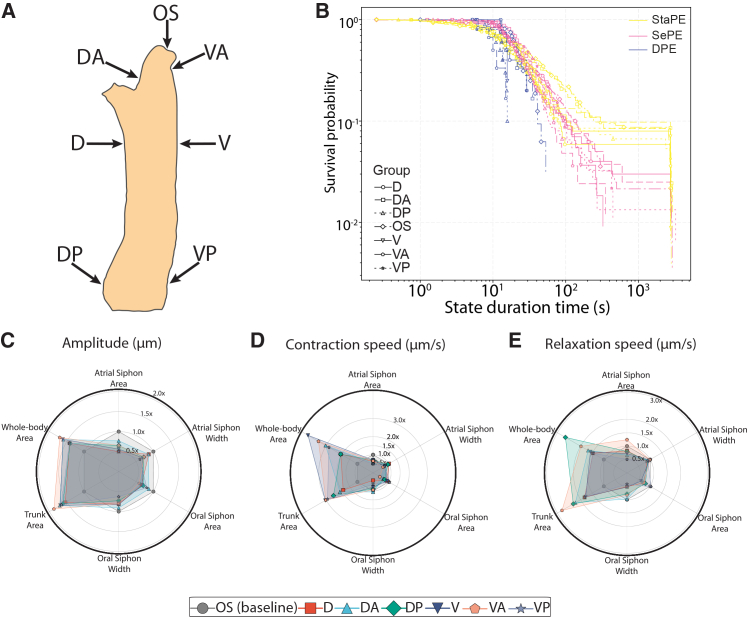
Region-specific behavioral responses to mechanical stimulation in adult *Ciona* (A) Schematic of the six body regions targeted for poke stimulation: oral siphon (OS), dorsal (D), ventral (V), dorsal anterior (DA), dorsal posterior (DP), ventral anterior (VA), and ventral posterior (VP). Please see Table S2 for a summary description of body part movements in response to the mechanical stimulation of these regions. (B) Survival curves of behavior state durations across the different stimulation sites. (C–E) Plots quantifying the percentage change relative to controls (no stimulus) of various postural and temporal features in response to poke stimulation in different anatomical regions (oral, atrial siphons, and the trunk) of the *Ciona* adult. Polar plots showing the impact of poke stimuli at different anatomical locations for amplitude (C), contraction (D), and relaxation speed (E), Please see Table S2 for a summary description of body part movements in response to the mechanical stimulation of these regions. For all panels animals used were as follows (OS *n* = 28, D *n* = 7, V *n* = 8, DA *n* = 8, DP *n* = 10, VA *n* = 6, VP *n* = 7).

While the mechanosensitivity of the oral and atrial siphons has been demonstrated in previous studies,^56^^,^^57^ the mechanosensitivity of other regions of the adult *Ciona* body has not, to our knowledge, been systematically examined. We therefore asked whether mechanical stimulation at different anatomical sites would evoke behavioral responses, and whether these responses would vary depending on the region stimulated. To address this, we delivered mechanical poke stimuli to six defined body regions—D, dorsal anterior (DA), dorsal posterior (DP), V, ventral anterior (VA), and ventral posterior (VP)—and compared these responses to pokes directed at the canonical OS site near the oral siphon (Figure 4A; Table S2).

We first assessed whether the distribution of state durations showed site-dependent differences. Across all poke locations, StaPE and SePE displayed long-tailed duration distributions (Figure 4B, yellow and magenta lines, respectively). StaPE events were typically the longest, consistent with extended quiescent intervals, whereas SePE exhibited intermediate durations with gradual decay. In contrast, DPE events were uniformly short and declined rapidly across all poke sites (Figure 4B, blue lines). Although these general patterns were preserved across sites, several region-specific effects were evident. Stimulation of the V, VA, and DP regions produced shorter DPE events compared to OS stimulation (Figure 4B). SePE durations were broadly similar across all sites, including OS (Figure 4B). StaPE durations were reduced following stimulation at the D, VP, and DP sites (Figure 4B). Together, these results indicate that although the overall structure of the state-duration distributions is conserved, the time spent in each behavioral state is modulated by the anatomical location of mechanical input.

We next analyzed whole-body and regional kinematics, focusing on trunk, oral siphon, and atrial siphon movements. The amplitude of oral and atrial siphon area and width decreased at all poke sites relative to OS stimulation, indicating a general reduction in siphonal opening dynamics regardless of location (Figure 4C). In contrast, whole-body and trunk area amplitude increased for all sites, with the strongest expansion observed following VA stimulation. Contraction speeds of the oral and atrial siphons were comparable across all sites relative to OS (Figure 4D). However, whole-body and trunk contraction speeds were consistently elevated across all poke sites, with the largest increase observed following V stimulation, followed by VA, VP, and DA (Figure 4D). Whole-body relaxation speed was greatest after DP stimulation, whereas VA stimulation evoked elevated trunk relaxation speed and faster atrial siphon relaxation relative to OS stimulation (Figure 4E).

Overall, these results demonstrate that the mechanical stimulation of different anatomical regions produces distinct sensorimotor responses in adult *Ciona*, affecting both the duration of behavioral states and the kinematics of specific body structures (Table S2). This regional specificity suggests that mechanosensory inputs are differentially integrated across the adult body, leading to site-dependent modulation of motor output.

### *Ciona* adults elicit distinct behavioral responses to chemosensory cues

**Figure 5:**
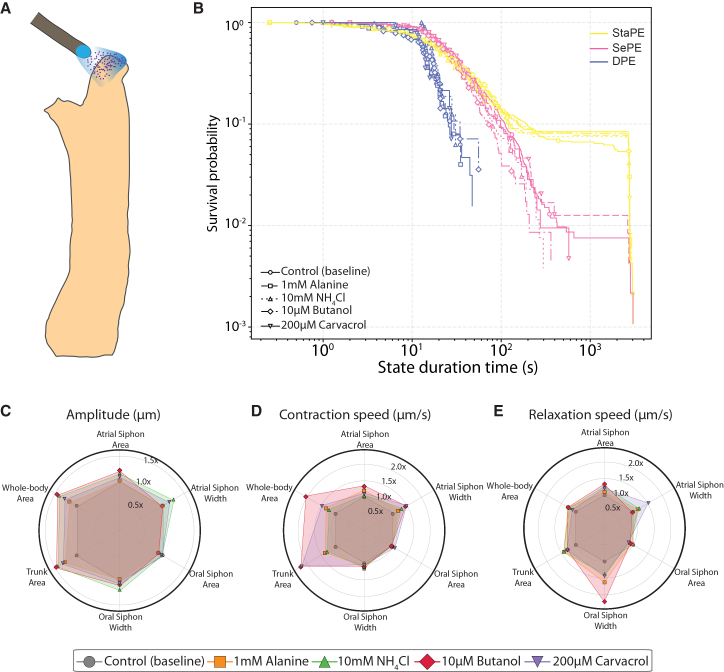
Specific behavioral responses to chemosensory stimulation in adult *Ciona* (A) Schematic of the chemosensory stimulation. Adult *Ciona* were exposed to four chemical cues: 1 mM alanine, 10 mM NH_4_Cl, 10 μM butanol, and 200 μM carvacrol, while artificial seawater (ASW) perfusion served as a control condition. (B) Survival curves of behavioral state durations across chemosensory conditions. (C–E) Quantification of the percentage change relative to ASW controls of postural and temporal features in response to chemosensory stimulation. Polar plots summarize the effects of different chemical cues on response amplitude (C), contraction (D), and relaxation speed (E) in adult *Ciona*. Please see Table S2 for a summary description of body part movements in response to chemical cues. Animals used were Control *n* = 17, 1 mM alanine *n* = 19, 10 mM NH_4_Cl *n* = 19, 10 μM butanol *n* = 17, and 200 μM *n* = 17.

We next asked whether adult *Ciona* are capable of detecting chemical stimuli and generating distinct behavioral responses. Previous work has shown that *Ciona* larvae can sense a variety of compounds, including terpenes, ammonia, short-chain alcohols, and amino acids.^29^^,^^34^ To test whether the adult stage retains chemosensory abilities, we developed a simple perfusion protocol in which chemical stimuli were delivered near the oral siphon (Figure 5A), a region likely to contain chemosensory cells based on the expression of markers related to the vertebrate olfactory placode.^74^ We exposed animals to four chemical cues: 1 mM alanine, 10 mM NH_4_Cl, 10 μM butanol (which are attractive cues for *Ciona* larvae), and 200 μM carvacrol (which is a repulsive cue for *Ciona* larvae)^29^ and compared their responses to artificial sea water (ASW) perfused controls (Table S2).

We first explored whether the distribution of state durations showed chemical stimulus-dependent differences. Across all chemical cues, StaPE and SePE states showed long-tailed duration distributions (Figure 5B, yellow and magenta lines, respectively). StaPE events were the longest, while SePE state events exhibited intermediate durations. Similar to mechanical stimuli, DPE events were short and declined rapidly across all chemical stimuli (Figure 5B, blue lines). While these general patterns were conserved across the four chemical cues, several stimulus-specific effects were apparent. Stimulation with butanol produced shorter SePE events compared to ASW controls, while carvacrol produced longer SePE events (Figures 5B, S5A–S5J, and S6A–S6E).

We next analyzed whole-body and regional kinematics. All chemicals increased the amplitude of whole-body and trunk area changes, with butanol producing the largest enhancement (Figure 5C). Ammonium chloride elicited the strongest increase in atrial and oral siphon width amplitude (Figure 5C). Multiple chemicals also increased contraction amplitudes of the whole body, trunk, and atrial siphon, with butanol and carvacrol producing the most pronounced effects (Figure 5C). These two compounds similarly generated the strongest increases in atrial siphon contraction speed (Figure 5D). Relaxation dynamics were also chemically modulated. Carvacrol increased the relaxation speed of the atrial siphon width, whereas butanol produced a particularly strong increase in oral siphon relaxation speed (Figure 5E). Together, these results demonstrate that adult *Ciona* respond robustly to a range of chemical cues, exhibiting distinct and compound-specific changes in behavioral states, body, and siphonal kinematics (Table S2). These findings provide evidence that *Ciona* adults possess functional chemosensory pathways capable of driving diverse motor outputs.

### *Ciona* adult behavior repertoire can be modeled in terms of states and transitions, which are modulated by mechanical and chemical stimuli

**Figure 6:**
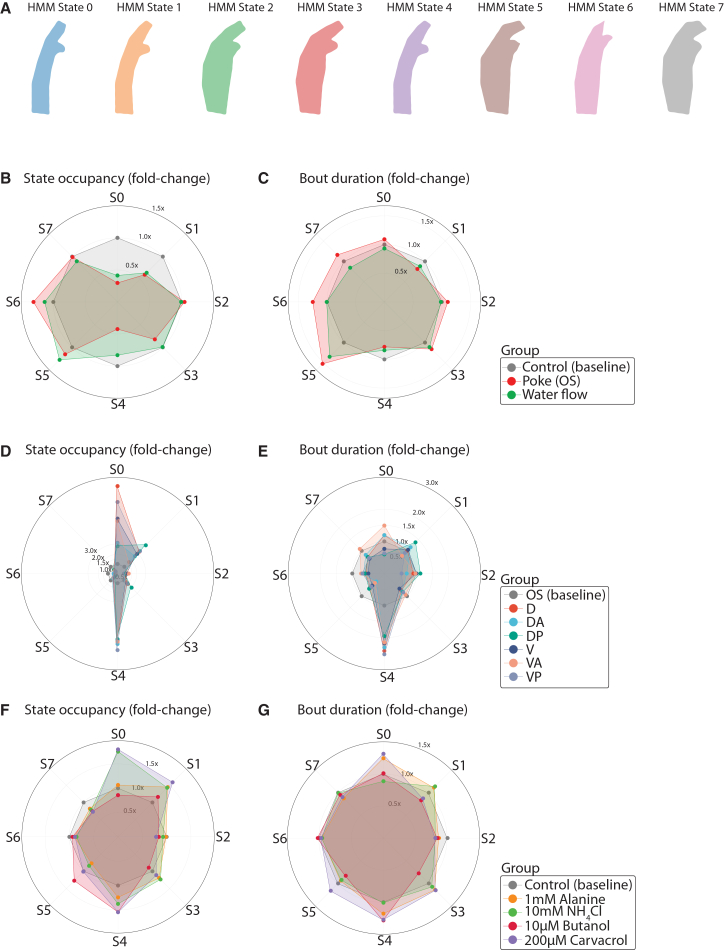
HMM analysis reveals that behavioral state usage and transitions in *Ciona* adults are modulated by mechanical and chemical cues (A) Representative reconstructed shapes corresponding to each HMM-inferred behavioral state (S0–S7), Please see Table S1 for a summary description of body part movements for each HMM-inferred behavioral state. (B–C) Radar plots show fold-change in state occupancy (B) and bout duration (C) under control (baseline), Poke of the OS, and water flow conditions. Number of animals used: control *n* = 52, poke OS *n* = 28, and water flow *n* = 23. (D–E) Region-specific mechanical stimulation induced changes in occupancy (D) and bout-duration. Number of animals used per condition: OS *n* = 28, D *n* = 7, V *n* = 8, DA *n* = 8, DP *n* = 10, VA *n* = 6, VP *n* = 7. (E) relative to baseline (OS poke). (F–G) Chemical stimulus-induced modulation of behavior states. Radar plots show fold changes in state occupancy (F) and bout duration (G) relative to artificial seawater controls (baseline). (Control *n* = 17, 1 mM alanine *n* = 19, 10 mM NH_4_Cl *n* = 19, 10 μM butanol *n* = 17, and 200 μM Carvacrol *n* = 17).

We next sought to expand our analysis by examining the organizational principles underlying the repertoire of behaviors expressed by adult *Ciona*. Assuming that adult *Ciona* behavior is modular and operates across multiple timescales, we modeled the full behavioral dataset using a HMM. An eight-state model (S0–S7) was implemented across all experimental conditions, with each state corresponding to a distinct body configuration with gradual increase of contraction intensity (Figure 6A; Table S1). These states served as the foundation for subsequent comparisons, quantified through state occupancy (fraction of total time spent in each state) and bout duration (median dwell time per visit).

Both mechanical stimulation (poke) and water flow induced clear, state-specific reweighting of the HMM repertoire (Figures 6B and 6C). Relative to baseline, mechanical poke increased occupancy of S5 and S6 while decreasing all other states except S2, which remained comparable to controls (Figure 6B). Water flow stimulation produced a partially overlapping but distinct signature: States S5 and S6 were again upregulated, with S5 showing a stronger increase than in poke-stimulated animals, whereas all remaining states were reduced or unchanged relative to controls (Figure 6B). Poke increased bout duration across nearly all states except S1 and S4, with the strongest effects observed for S5, followed by S6 and S7 (Figure 6C). Notably, states S5–S7 correspond to contracted body configurations with closure of the oral and/or atrial siphon, suggesting that mechanical stimulation promotes defensive or withdrawal-like postures. Bout durations under water flow were elevated only in S5 and S3; all other states exhibited reduced or baseline-level durations (Figure 6C). To examine how the poke of the oral siphon and water flow influence behavioral state dynamics, we quantified transition probabilities among the HMM-derived states across the three conditions, which we visualized using chord diagrams and transition probability matrices (Figures S7A–S7F). Under control conditions, transitions were broadly distributed, with no single state or transition dominating the behavioral repertoire (Figures S7A and S7B). Relative to control, poke strongly rerouted transitions originating from S0 toward S1 (S0→S1: 0.18→0.40) while suppressing S0 outflow to S4/S5/S7 (all 0.00 under poke) (Figures S7C and S7D). Poke of OS also reduced self-transitioning of S4 probability (S4→S4: 0.36→0.09) and increased transitions from several states into S6 (e.g., S1→S6: 0.21→0.28; S2→S6: 0.18→0.32; S3→S6: 0.26→0.33; S4→S6: 0.18→0.31), indicating a stimulus-driven bias toward S6. In contrast, water flow redirected S0 primarily to S1 and S5 (S0→S1: 0.18→0.25; S0→S5: 0.06→0.25) while decreasing S0→S6 (0.24→0.12). Water flow reduced self-transitioning S7 probability (S7→S7: 0.41→0.34) and increased coupling of S7 with S6 (S7→S6: 0.27→0.31) (Figures S7E and S7F). Across both stimuli, transitions into S6 were elevated, but poke OS stimulation produced stronger and more widespread effects (Figure S7).

Taken together, these results indicate that both mechanical and hydrodynamic stimuli engage a shared subset of contraction-associated states, but with distinct temporal dynamics and state switching, implying that *Ciona* can discriminate stimulus modality through differential modulation of state persistence.

We then examined the impact of poke location relative to the OS site, which we considered as a baseline mechanical stimulation site. All alternative poke locations showed reduced occupancy of S5–S7 relative to OS stimulation (Figure 6D). In contrast, all locations showed an increase in S0 occupancy, with the D, V, VP, and VA positions exhibiting the largest increases, and State S4 was also consistently upregulated across all positions. States S1 and S3 showed modest increases for all locations, with DP producing the strongest effect (Figure 6D). A pronounced increase in bout duration for S4 was observed for all sites compared with OS stimulation, while position VA also increased bout duration for S0, and position DP increased bout duration for S1 (Figure 6E). Reduced bout durations were observed for S5-S6 for all sites compared to OS, implying reduced time allocated to contracted postures when stimulations occurred more distally from the OS.

To further resolve the spatial specificity of mechanosensory modulation, we compared transitions of HMM states between poke at the OS position and the other six positions across the adult body (Figures S7C, S7D, and S8A–S8L). In contrast to OS stimulation, which generated multiple transitions from S4 and strong convergence of transitions from other states into S6 (Figures S7C and S7D), each of the six alternative poking sites instead increased S4 self-persistence (S4→S4), resulting in a more stable S4 state across D, V, and anterior, posterior perturbations (Figures S8A–S8L). All non-OS stimulation sites showed reduced transition into state S6, indicating a reduced adoption of the S6 state (Figures S8A–S8L). Differences were most obvious (as compared to OS poking) in animals poked on the VA area, followed by VP and D stimulations (Figures S7A, S7B, S9A, S9D, S9H, S9I, S9K, and S9L). In contrast, DA poking resulted in the smallest change relative to the OS HMM state transitions pattern, despite the reduced transitions from all states to the S6 state (Figures S8B and S8E). In addition, poking position influenced S0 transitions: OS shifted S0 toward S1 and S6, whereas D, VA, and VP shifted S0 into S4, and DP switched S0 primarily into S1 (Figures S7C, S8A, S8D, S8H, S8I, S8K, and S8L). Our work suggests a spatial specificity in mechanosensory encoding, whereby different anatomical regions trigger systematically different patterns of behavioral state transitions.

**Figure 7:**
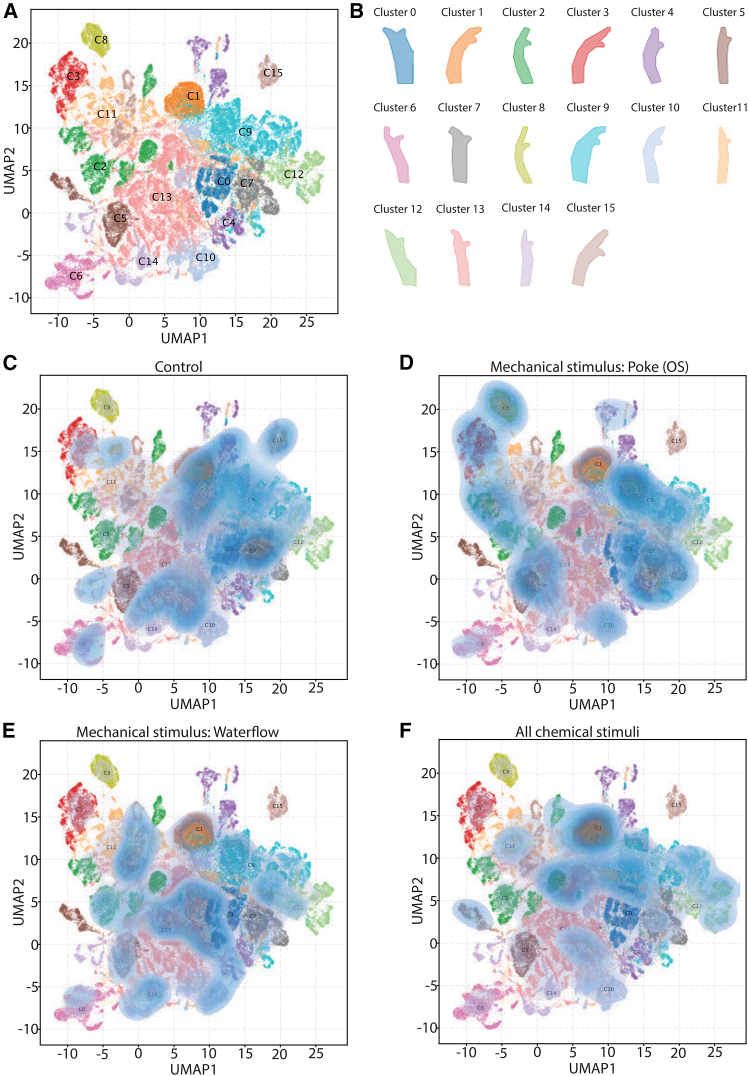
Low-dimensional spatiotemporal embedding reveals stereotyped postural modes in sessile adults *Cio*na (A) Two-dimensional UMAP embedding of postural features derived from the first five postural principal components. Points are colored by postural cluster identity inferred from Gaussian mixture modeling (GMM; K = 16). (B) Representative reconstructed body shapes for each postural cluster. (C–F) Kernel density estimates (KDEs) showing the occupancy of the behavioral space under control. (*n* = 52) (C), oral siphon poke (*n* = 28), (D), water flow (n = 23), (E), and pooled chemical stimuli (F) conditions (*n* = 89 in total), Colored points indicate postural cluster identities overlaid on the density contours (blue).

Finally, we assessed the effects of different chemical stimuli on state occupancy and bout duration relative to artificial seawater controls. 200 μM carvacrol and 10 mM NH_4_Cl increased occupancy of S0, while 1 mM Alanine, 200 μM carvacrol, and 10 mM NH_4_Cl increased occupancy of S1 (Figure 6F). State S4 was upregulated by 10 μM butanol and 200 μM carvacrol, while S5 showed only a moderate increase in occupancy in response to 10 μM butanol (Figure 7F). 200 μM carvacrol produced the strongest and most widespread increases in bout duration among all chemicals tested and was the only stimulus that increased S5 bout duration (Figure 7G). 10 μM butanol also increased bout duration in S4 (Figure 6G). Comparisons of HMM state-transition probabilities showed that each chemical treatment modified behavioral dynamics in a distinct manner relative to ASW controls (Figures S9A–S9J). 1 mM Alanine resulted in a subtle shift in transition probabilities, characterized by increased persistence of select states (notably S2 and S6) and enhanced transitions into S6 from multiple upstream states, consistent with a modulatory effect on ongoing behavioral activity (Figures S9B and S9E). In contrast, stimulation with 10 mM NH_4_Cl elicited a different HMM state transition architecture, amplifying S4 self-transitions and increasing transition probabilities into S4 from S0, S1, and S3 states (Figures S9C and S9F). Stimulation with 10 μM butanol also altered the transition probabilities, but in a pattern distinct from NH_4_Cl. Butanol increased transitions into S5 and S6 while simultaneously reducing S4 persistence (Figures S9G and S9I). Finally, 200 μM carvacrol produced the most extensive differences in transition probabilities relative to ASW controls, strongly increasing S7 persistence and upregulating transitions into both S6 and S7 (Figures S9H and S9J). These results indicate that chemical cues engage behavioral states distinct from those induced by mechanical or hydrodynamic stimulation, with carvacrol eliciting particularly prolonged responses suggestive of aversion or stress-associated postural patterns. However, similarly to mechanical and hydrodynamic stimulation, chemical stimulation induces cue-specific reorganization of the behavioral state transitions, revealing the sensitivity of these HMM-defined states to chemosensory cues.

### Low-dimensional spatiotemporal embedding reveals stereotyped actions in sessile adults

In earlier sections of our manuscript, we considered *Ciona* adult behavior as a sequence of discrete actions and states that we represented in ethograms. An assumption at the core of this approach is that *Ciona* adults perform behavioral actions in a way that allows for their discrete classification.^61^ However, we wanted to leverage a complementary approach aiming to reveal new structures in our behavioral data that may have been hard to detect initially by relying on human observations. To this end, we employed a spatiotemporal embedding approach^19^ to reveal stereotyped behaviors exhibited by *Ciona* adults using unsupervised segmentation.

For this approach, we clustered shape trajectories in a lower-dimensional “behavioral space” based on PCA-transformed EFD amplitudes and visualized it with uniform manifold approximation and projection (UMAP). Subsequently, this embedding, which we can consider as the adult behavioral space, was sub-clustered using the hierarchical density-based spatial clustering of applications with noise (HDBSCAN) and Gaussian mixture models algorithms (Figure 7A). We identified 16 clusters corresponding to different stereotyped behavioral modules (Figure 7B; Table S1). Interestingly, this approach allowed us to identify stereotyped behavioral modules that are hard to discern by manual approaches. For example, cluster 11 corresponded to animals with elongated siphons whose aperture was closed. Another interesting example is cluster 9, where we observe a retraction of the closed atrial siphon but not the oral siphon.

We generated Kernel density estimation (KDE) plots for control unstimulated animals (spontaneous behaviors only) as well as for animals that were presented either with a poke OS, a water flow stimulus, or merged chemical stimuli (Figures 7C–7F). Far from being uniformly distributed across the behavioral space, the probability density contains several resolved local maxima. The locations of these maxima provide a potential representation for the stereotyped behaviors that the *Ciona* adults perform. Unstimulated control animals visited most regions of the behavioral space, showing a large diversity of behavioral modes (Figure 7C). Animals stimulated with a mechanical poke on the oral siphon showed fewer maxima, indicating a more limited use of behaviors in response to mechanical poke perturbation (Figure 7D). Interestingly, local maxima were observed in clusters which were indicative of closing of both the atrial and oral siphons and/or a full body contraction (e.g., clusters 4, 9, 10, and 11) (Figure 7D), consistent with the strong response we see in the videos. Water flow probability density was concentrated in partially distinct clusters compared to OS poked animals. These clusters represented behaviors where the atrial and/or oral siphons were closed, but the body was not contracted (e.g., clusters 0 and 14) or both siphons were open yet moderately withdrawn toward the trunk (e.g., cluster 11) (Figure 7E), consistent with the responses we have observed in the original movies.

We also examined the distribution of pooled chemical stimuli, which showed patterns distinct from both oral siphon pokes and water-flow responses. Clusters with enriched densities included clusters 1 and 13, where the atrial siphon is partially closed and/or withdrawn, as well as cluster 12, where the oral siphon is closed but the atrial siphon is partially withdrawn without being fully closed (Figure 7F).

Finally, we quantified the mean individual time an adult *Ciona* spent on each cluster/behavioral module (Figure S10). Unstimulated animals (control) predominantly occupied cluster 13, followed by cluster 11, 10, and 9. These clusters corresponded primarily to relaxed postures with an elongated trunk and either both or one of the siphons fully extended and with open aperture. Beyond the four dominant behavioral clusters, the control animals exhibited a short amount of time in residual clusters of the behavioral space. Mechanically stimulated animals shifted toward increased occupancy of clusters 10 and 11 compared to the control, indicating prolonged siphon closure or partial closure associated with an oral siphon stimulation. In the case of water flow, animals dwell for a longer time in behavioral clusters 0 and 2, and reduced time in clusters 9 compared to control and mechanically stimulated animals. These clusters suggest that water flow enhances behavioral transitions reflecting increased trunk dynamics and active modulation of atrial siphon aperture to regulate flow through the branchial basket. Chemically stimulated animals displayed elevated time spent in clusters 1, 9, and 12 that correspond to clusters with partially closed siphons, and thus, less time spent with open siphons, potentially restricting flow and chemical entry. These results support that adult *Ciona* have stimulus-specific occupancy patterns.

## Discussion

Here we present a quantitative framework for characterizing the spontaneous and stimuli-evoked behaviors exhibited by adult *Ciona intestinalis* individuals. Compared to past behavioral studies on adult ascidians, this study benefits from cutting-edge tracking and analysis tools, leading to a data-rich and highly quantitative and detailed characterization of the behavioral repertoire of adult *Ciona intestinalis*. In our approach, posture information can be leveraged to detect subtle behaviors that would not have been discernible from mere qualitative observations or the use of other forms of tracking (e.g., centroid-based). Thus, a major advancement of this work is the use of the animal contour (outline), which was extracted in a high-throughput and reliable manner by using a trained deep neural network.^64^ In this way, we measured morphometric features such as siphon contraction and relaxation speeds, siphon aperture size, and body angle. Importantly, we have derived a dimensionality-reduced representation of adult *Ciona* postures (i.e., eigencionas). We demonstrated that five eigencionas can be combined in different fractions to reconstruct almost the entirety of behaving adult *Ciona* postures. This likely reflects fundamental constraints on *Ciona* adult behavior, where all behaviors of the adults explore different regions of the same shape space. This is a phenomenon observed in several organisms including *C. elegans*,^19^^,^^75^ *D. melanogaster*^76^ and *D. rerio*.^77^^,^^78^ Having established a compact and common basis for representing *Ciona* shapes in two key phases of the biphasic life cycle the larva^19^ and adult stages (this study), future studies can leverage this resource in the context of high-throughput behavioral phenomics screens (e.g., to study the impact of anthropogenic pollutants such as neuroendocrine disruptors on marine organisms^79^) or detailed neurobiological studies. Past and ongoing efforts to map the developmental origin^52^^,^^80^ and innervation patterns of the adult nervous system^60^ can now be combined with an in-depth study of the adult postural dynamics.

In parallel to employing body part kinematics, postural dynamics, and eigencionas, we have also implemented a spatiotemporal mapping approach similar to what has been employed in mapping complex behavioral responses in mice, flies, and more recently in *Ciona* larvae.^81^^,^^82^^,^^83^ Here we present 16 annotated clusters of different behavioral modes that segregate to discrete regions of the behavioral space map. An important conclusion of our work is that the behavioral responses we observed in response to different mechanical cues (poke and water flow) do not result in global changes in the underlying spatiotemporal structure of *Ciona* adult behavioral space, but rather they arise from the selective use of behavioral modules and changes in the amount spent in each behavioral module. This suggests that the *Ciona* adult brain can select which individual behavioral modules it will use (and for how long it will use them) to generate responses to novel situations such as the presentation of mechanical stimuli.

In this study, we made the surprising discovery that *Ciona* adults, which have long been described as passive filter feeders with a rather simple nervous system,^57^^,^^60^ can exhibit different behavioral states. These states are distinguished from each other by various postural and motion features, including but not limited to the opening and closing speed and amplitude (aperture) of the oral and atrial siphons and the rate and extent of contraction of the trunk (i.e., how fast and how much they shrink and relax their body). Importantly, they can persist for different lengths of time and exhibit distinct transition probabilities. We have found that transitions between behavioral states can be induced by external stimuli (water flow and poke). Transitions between states can also occur in control animals. These may reflect responses to ambient light, subtle changes in temperature, or solid particles that are siphoned into the animals.

In other model organisms such as *C. elegans,* the duration of behavioral states is influenced by factors such as sensory cues and the animal’s internal state.^69^^,^^84^^,^^85^ In the case of *Ciona* adults, we demonstrate that sensory cues can alter the duration of states and especially of the DPE state. Interestingly, a poke stimulus that induces a very strong and lasting DPE state is followed at a higher probability (compared to the no stimulus condition) by StaPE state (i.e., a low activity state). While this may be a surprising observation in the first instance, behavioral challenges can cause animals to switch from active to passive strategies for coping with effort expenditure during stress.^86^ How might these states be modulated at the molecular and cellular levels? The oral and atrial siphons, which show a strong modulation based on the behavioral state of the *Ciona* adult, are innervated by a series of anterior and posterior peptidergic nerves.^60^ Given the richness of neuropeptides present in the adult nervous system,^87^^,^^88^^,^^89^^,^^90^^,^^91^ it is plausible that peptidergic volume transmission is involved in modulating the duration and features of the behavioral states exhibited by *Ciona* adults, akin to what has been observed in other nervous systems across the tree of life.^92^^,^^93^^,^^94^^,^^95^^,^^96^

How is it that adult *Ciona* can distinguish between these two different mechanical cues? One possibility is that *Ciona* adults use distinct populations of sensory cells to detect these different mechanical cues. *Ciona* is equipped with 75–100 cupular sense organs, which are found in the lining of the atrial siphon.^43^^,^^97^ The localization of the cupular organs close to the atrial siphon indicates that they may monitor water flow; however, to date, we lack supportive functional evidence. Conversely, the oral siphon of *Ciona* adults is equipped with a row of secondary sensory cells that form the coronal organ.^98^^,^^99^ In the colonial tunicate *B. schlosseri,* these cells are thought to mediate the oral siphon stimulation test (an acute waterjet stimulus),^100^ while in the solitary ascidian *Corella inflata* they respond to touch by a needle.^56^ Thus, it is conceivable that the coronal organ is responsive to poke stimuli. The ability to distinguish water flow stimuli from other mechanical cues is of profound importance, given that most sessile filter feeders depend on ambient water flow to replenish their food supplies and to remove waste products.^101^ Similarly, the ability to detect acute mechanical stimuli (e.g., a poke) that may correspond to a benthic predator, such as sea urchin, crabs, sea stars, and others, is equally important for the survival of juvenile and adult ascidians.^102^^,^^103^^,^^104^^,^^105^

Our findings show that adult *Ciona* exhibits clear regional differences in mechanosensory responsiveness, indicating that the body surface is not uniformly sensitive to mechanical input. Although siphon mechanosensitivity is well established, our results demonstrate that the stimulation of multiple additional regions reliably evokes distinct behavioral and kinematic responses. State-duration analyses revealed that StaPE and SePE maintain long-tailed distributions across all stimulation sites, suggesting these states represent stable behavioral states. In contrast, DPE durations were consistently brief and showed further shortening at specific regions (V, VA, DP), indicating that some body areas preferentially trigger fast, transient responses. Reductions in StaPE duration at D, VP, and DP sites further highlight region-dependent modulation of quiescent behavior. Kinematic analyses supported this spatial specificity. While siphon opening uniformly decreased across all sites—consistent with a generalized protective reflex—trunk and whole-body responses varied substantially with poke location. VA and V stimulation elicited the strongest increases in expansion and the fastest contraction speeds, whereas DP and VA stimulation produced the highest relaxation speeds. These patterns suggest that distinct mechanosensory “hotspots” shape localized motor outputs.

Together, these results indicate that adult *Ciona* integrates mechanosensory input in a spatially organized manner, engaging a shared set of behavioral modules but modulating their timing and magnitude depending on stimulus location. Previous studies leveraging a transgenic reporter labeling peptidergic neurons have reported putative innervation patterns along the D and V sides of the animal, which may receive mechanosensory input and relay that information to the neural complex.^60^^,^^106^ Similarly, studies in other ascidians have provided evidence for the presence of sensory neurons in the periphery that may mediate diverse sensory responses.^57^^,^^107^ The regional tuning likely reflects the functional roles of different body zones and provides a framework for future work linking mechanosensory regions to underlying neural circuitry.

The presence of putative chemosensory cells in adult tunicates has been proposed for the thaliacean *Thalia democratica*^108^ and the appendicularian *Oikopleura dioica,* which expresses the markers Pitx and Six 3/6 in the primordial ciliary funnel;^109^ however, behavioral responses to chemical stimuli in adult tunicates have not been demonstrated to date. Our results revealed that adult *Ciona* retain functional chemosensory abilities and exhibit clear, stimulus-specific behavioral modulation. The effects of chemical stimuli were more pronounced in terms of kinematic changes, suggesting that each chemical cue elicits a unique motor signature. The precise identity and location of putative chemoreceptor cells is currently unknown, but they could be located in the oral siphon, since the oral siphon primordium in the larvae expresses Six1/2, Six3/6, and Pitx, well-established markers of the olfactory placode in vertebrates.^110^ Future molecular, ultrastructural, and functional studies will reveal the identity of these chemosensors.

The HMM-based analysis of the adult *Ciona* behavioral repertoire revealed that both mechanical and hydrodynamic stimuli recruited a shared subset of contraction-associated states, yet they did so with distinct weighting and temporal organization. These stimulus-dependent redistribution patterns, together with the reduction in transition diversity observed under poke, point to a streamlined defensive behavioral response characterized by increased engagement of contraction states. Water flow produced a partially overlapping but weaker contraction signature, with reduced dwell times for most states and a distinct reorganization of transitions compared to OS poke stimulation. The differences in both state persistence and transitions between mechanical and hydrodynamic stimuli indicate that *Ciona* discriminates stimulus modality not through unique states but through the differential modulation of transitions and dwell times within a shared behavioral repertoire. Mechanosensory stimulation of multiple anatomical regions further demonstrated that *Ciona*’s behavioral state dynamics encode not only stimulus modality but also stimulus location. The additional finding that chemical stimuli altered state occupancy and transitions in a cue-specific manner suggests that the adult *Ciona* behavioral repertoire is modular and stimulus-dependent, with modality- and location-specific postural states and transition dynamics. This organization enables a relatively simple nervous system to flexibly encode diverse environmental cues.

To date, reports on the behavior of invertebrate sessile benthic organisms have been sparse and have mostly focused on *Poriferans* (i.e., sponges)^30^^,^^31^ and abyssal anemones.^111^^,^^112^ One of these studies has used convolutional neural networks to study sponge behavior over time.^30^ Benthic communities are linked with various processes occurring at the sea surface, despite the vertical depth distance that can separate the two. Many studies have focused on highly motile fauna, while the sessile fauna of benthic environments has largely been ignored despite several observations that sessile organisms can show dynamic lifestyles, showing both spontaneous and rhythmic behaviors.^30^^,^^31^ We believe that our approach and investigation of behavioral states can be extended to other ascidian adults as well as other sessile benthic organisms such as barnacles and articulated coralline algae.

Finally, from an evolutionary perspective, our work on the behavior of *Ciona* adults allows us to get a glimpse of the possible behavioral repertoire of extant sessile epibenthic organisms. Our study may be particularly relevant for mid-Cambrian tunicates such as *Megasiphon thylakos,* which have both circular and longitudinal musculature such as modern ascidians.^13^ It is thus conceivable that together with the establishment of the fundamental components of the modern tunicate body plan, the behavioral traits of tunicates had already evolved almost 500Mya ago.

### Limitations of the study

We implemented two-dimensional (2D) video recording to analyze body kinematics and posture dynamics of adult *Ciona intestinalis*. Although 2D analysis can approximate three-dimensional (3D) behavior—particularly for movements occurring mainly in a single plane,^113^^,^^114^^,^^115^ it is limited by camera-angle dependence. In contrast, 3D tracking methods eliminate this issue and provide more accurate pose estimation and kinematic data. However, 2D tracking remains popular due to the complexity and cost of 3D systems. As tracking hardware becomes more affordable and 3D analysis tools more advanced, we expect opportunities to apply 3D pose estimation to tunicates, including adult *Ciona*. Additionally, recent deep learning approaches that infer 3D poses from 2D data,^116^ offer a promising alternative to full 3D tracking systems.

In this study, we tested a broad range of stimuli and found that adult *Ciona* exhibit a surprisingly robust capacity to generate behavioral responses to diverse mechanical and chemical cues. A limitation of our current assays is the reliance on manual stimulus delivery, which can introduce variability in stimulus strength, timing, and duration. Future work will benefit from the development of automated stimulus-delivery systems that provide more precise and standardized stimulation of Ciona adults and can dynamically adjust stimulus strength to the characteristics of each individual.

In addition, while we selected animals of similar dimensions in terms of length and width, we cannot exclude the possibility that other more subtle differences (e.g., difference in dry weight) may influence their perception of the stimuli we delivered and thus introduce variability. The future use of age and size-matched adults cultured under laboratory conditions may help address this limitation.

## Resource availability

### Lead contact

Further information and requests for resources and reagents should be directed to and will be fulfilled by the lead contact, Marios Chatzigeorgiou (marios.chatzigeorgiou@uib.no).

### Materials availability

This study did not generate any new materials.

### Data and code availability

• Raw video data have been deposited in Zenodo. "Zenodo: https://doi.org/10.5281/zenodo.16608643, https://doi.org/10.5281/zenodo.16614277, https://doi.org/10.5281/zenodo.19742998".
• The DeepLabCut based DNN trained to recognize landmarks on Ciona intestinalis adults has been deposited in Zenodo together with the training dataset. "Zenodo: https://doi.org/10.5281/zenodo.16910611".
• All original code has been deposited at Zenodo: "Zenodo: https://doi.org/10.5281/zenodo.19742781 and will be publicly available as of the date of publication. Code to analyze behavioral data is already available here: "Github: https://github.com/ChatzigeorgiouGroup/Ciona_adult_behavior".
• Any additional information required to reanalyze the data reported in this paper will be made available from the lead contact upon request.

## Acknowledgments

We would like to thank members of the Chatzigeorgiou lab for valuable feedback on the manuscript. We acknowledge funding from the 10.13039/501100005416Research Council of Norway: 339399 (M.C.) and 335582 (M.C.).

## Author contributions

Conceptualization: O.T., S.N., and M.C.; methodology: O.T., S.N., and M.C.; data curation: O.T. and S.N.; investigation: O.T. and S.N.; visualization: O.T. and S.N.; resources: M.C.; funding acquisition: M.C.; project administration: M.C.; supervision: M.C.; writing – original draft: O.T., S.N., and M.C.; writing – review and editing: O.T., S.N., and M.C.

## Declaration of interests

The authors declare no competing interests.

## Declaration of generative AI and AI-assisted technologies in the writing process

During the preparation of this manuscript, Microsoft Copilot was used for polishing and grammar checking of the manuscript text. All output text was critically reviewed and edited, and the authors take full responsibility for the final content.

## STAR★Methods

### Key resources table

**Table:** 

REAGENT or RESOURCE	SOURCE	IDENTIFIER
**Chemicals, peptides, and recombinant proteins**		

Carvacrol	Sigma	282197-10G
Ammonium chloride	Alfa Aesar	A15000
L-Alanine	Sigma	A7469-100G
1-Butanol	Sigma	B7906-500 ML

**Deposited data**		

Raw videos of C. intestinalis adults	https://doi.org/10.5281/zenodo.16608643https://doi.org/10.5281/zenodo.16614277 https://doi.org/10.5281/zenodo.19742998	N/A
DLC DNN trained to recognize *C. intestinalis* body parts and training data	https://doi.org/10.5281/zenodo.16910611	N/A

**Experimental models: Organisms/strains**		

*Ciona intestinalis* wild caught (formerly *Ciona intestinalis* type B)	Sotra, Vestland, Norway	N/A

**Software and algorithms**		

Open Broadcaster Software (OBS) Studio	https://obsproject.com/	Version: 31.0.3
DeepLabCut	Lauer et al.^64^	N/A
pyefd	https://github.com/hbldh/pyefd	Version: 1.6
pywt	https://github.com/PyWavelets/pywt	Version 1.6
Matplotlib	https://github.com/matplotlib/matplotlib	Version 3.8
numpy	https://github.com/numpy/numpy	Version 1.26
Scikit-learn	https://github.com/scikit-learn/scikit-learn	Version 1.5
UMAP	https://github.com/lmcinnes/umap	Version 0.5
Custom code for *C. intestinalis* adult behavior analysis	https://doi.org/10.5281/zenodo.19742781	N/A

### Experimental model and study participant details

We harvested wild adult *C. intestinalis* (*Ciona* intestinalis Type B) from the following sites: Døsjevika (60.342564, 5.114036), Kleppholmen (60.185215, 5.152785), Klokkarvik (60.222616, 5.157331), Liaskjeret (60.294301, 5.132846) and Toftøyna (60.482154, 4.950136), Sotra, Vestland, Norway. Wild *Cionas* were transferred to a purpose-built facility at the Michael Sars Center, University of Bergen. We kept approximately 100 adults in 50L tanks with constant running filtered sea water (FSW) at 10°C to 12°C and provided constant light and daily food supply. The diet included several species of diatoms and brown algae to increase egg production and reduce spontaneous spawning.^18^ Wild adults were allowed to acclimatize in the indoor facility for at least 24 h before behavioral recording.

### Method details

#### Behavioral recording

On the day of recording, one or two adults were transferred to 54 L experimental glass tanks (60 cm × 30 cm) filled with FSW and 5 mL of algae mixture (1.4 mL *Chaetoceros calcitrans*, 1.4 mL *Rhinomonas*, 1.4 mL *Isochrysis* sp., 700 μL *Synechoccoccus* sp., from algal stock culture), and overhead lights (color temperature = 2700 K, Mira 26B dual flex arm, NANLITE, China) were provided. In the glass tank, the animal posterior region of the body (holdfast-end) was gently mounted vertically in a “mounting rack” located at the tank bottom. This mounting rack consisted of the top panel of a tube rack (Snap-Together canonical tube rack for 15 mL tubes, Heathrow Scientific, IL, USA) fixed on top of a plastic basket. The tube rack was covered with waterproof black spray paint to optimize the optical field. Additional weight was added to the basket allowing the rack to sink to the bottom of the tank as well as preventing movement during recording. The animals recorded were selected for their height and coverage of other organisms. The height of adult *Ciona* was 11.31 cm ± 2.62 cm after mounting. In cases where *Ciona* was covered by other organisms, such as algae or smaller *Ciona* that had settled on an adult, both oral and atrial siphon rims and cavities needed to be visible and differentiated from the lateral side. The choice of one or two animals recorded at once was based on their morphological curvature after mounting on the racks despite the provided overhead lights. Two animals were used if the angle of the mounted animals did not prevent full visualization of the neighboring animal during recording. The animals were mounted in the dorsoventral orientation according to the camera position for recording, allowing visualization of both siphons. The animals were recorded using Logitech HD Pro c920 (Logitech, Switzerland) controlled by Open Broadcaster Software (OBS) Studio (https://obsproject.com/). Data were recorded at 20 frames per second.

#### Mechanical and chemical stimuli exposure assays

After transfer and mounting in the rack, adult *Ciona* were acclimated in the experimental tank for 15 min without disturbance. This acclimation step was performed across all assays described hereafter. To investigate behavioral responses of adult *Ciona* to mechanical stimulation, we designed two complementary assays.

#### Assay 1: Mechanical stimulation at the oral siphon and water flow

Each animal was first recorded for 15 min under no-stimulus conditions. Following this baseline period, individuals were exposed to two types of mechanical stimuli:

**Poke stimulus:** A single poke to the outer region of the oral siphon (OS) using a borosilicate glass Pasteur pipette (weight: 2.98 g; tip radius: 750 μm). Behavior was recorded for an additional 15 min.

**Water-flow stimulus:** A directed water jet with a flow speed of 0.399 m/s was delivered 15 cm from the OS, corresponding to a cross-section shear rate of 2.85 s^−1^. Behavior was again recorded for 15 min. A total of 10 unique animals were used in this sequential protocol. To exclude potential carry-over effects between stimuli, we also performed additional experiments in which animals were exposed to a single stimulus type. For these experiments, animals were first recorded under control conditions and then exposed to the stimulus. For each stimulus type, we recorded at least 15 additional animals following this control - stimulus design. In addition, several animals were recorded exclusively under control conditions to further characterize baseline behavior.

#### Assay 2: Whole-body mechanical stimulation mapping

Each animal was first recorded for 15 min under control (no-stimulus) conditions. Subsequently, mechanical pokes were delivered at seven predefined body sites (see Figure 4A) in 15-min intervals, each stimulation and the subsequent 15 min intervals were video recorded: Oral Siphon (OS), Dorsal Anterior (DA), Ventral Anterior (VA), Dorsal (D), Ventral (V), Dorsal Posterior (DP), Ventral Posterior (VP).

The sequence of stimulated sites was alternated between animals to avoid sequence effects. Details on poking angle, indentation depth, and poke duration are provided in Table S3.

#### Chemosensory stimulation assays

Each animal was first exposed to a control stimulus (artificial seawater; ASW) with behavior recorded for 15 min. This was followed by sequential presentation of the following chemical stimuli (all dissolved in ASW): 1 mM alanine, 10 mM NH_4_Cl, 10 μM butanol, 200 μM carvacrol. For each stimulus we recorded the stimulus delivery and 15 min after that. In total, each animal was recorded for 75 min. Chemical stimuli were delivered via a Pasteur pipette positioned 4 cm from the OS at ∼5° angle, producing a flow speed of 0.0175 m/s and a cross-section shear rate of 0.438 s^−1^. Note that differences in the sample size for each chemical condition (between 17 and 19 animals) reflect cases where DLC output failed to pass quality check thresholds for a specific stimulus video.

After completion of the recording session, each adult was removed from the tank, stroked twice with a Pasteur pipette from posterior to anterior, and gently blotted with paper to remove excess surface water. Wet weight was then measured (mean: 12 g ± 2.65 g).

#### DeepLabCut model

The adult *Ciona* DLC model was trained to track 18 body points from the dorsal to the ventral and anterior to posterior positions based on external morphological characteristics, including the rims and cavities of the oral and atrial siphons, and along the trunk region. The adult DLC model was trained using 20 frames from 22 randomly selected videos (440 frames in total) for 200.000 iterations and three shuffles (other parameters were kept at default) with the ResNet50 ImageNet pre-trained weight architecture as the selected network architecture. 10 outlier frames from 34 videos were corrected following 100.000 iterations and three shuffles. The DLC model was characterized by train and test errors of 3.15 and 3.91 pixels, and 3.14 and 3.91 with 0.6 p-cutoff value. Mm-to-pixel conversion ratio was determined *in silico* to 0.254 mm/pixel. Data was generated for all videos, and the filtered tracking data were exported as CSV files and used for extracting features. Filtered coordinate tracking data (DeepLabCut outputs) were loaded from individual.csv files and merged after removal of confidence (likelihood) columns. Each animal’s body part coordinates (x and y) were split, resampled (1:5 temporal downsampling resulted in 4 Hz signal), and annotated based on experimental condition (control, water flow, or poke).

#### Extraction of supervised behavioral metrics

We computed six quantitative time series per animal: whole-body area (via 2D polygon surface), oral and atrial siphon opening widths (Euclidean distance between respective dorsal and ventral points), corresponding areas (defined by four-point contours), and a trunk area (polygon excluding siphon points). Only traces with ≥20% non-NaN data for all metrics were retained. Metric values were normalized by median, outliers filtered, missing data segments (≤20 frames) were interpolated. We applied seasonal decomposition (additive model, 50-frame periodicity) to isolate contraction trends from each metric. Trends were z-scored, extrapolated at the start (25-frame linear fit), and interpolated (≤200-frame gap fill). Initial and terminal valid points were marked to prevent boundary artifacts.

Contraction event detection on z-scored trends was performed using a smoothed signal (Savitzky-Golay filter), identifying contraction events via prominence based peak detection (minimum prominence: 0.7; minimum width: 5 frames). Detected events were categorized as “DPE” or 'SePE' events based on relative peak amplitude with 70% of maximum peak threshold. StaPE periods were defined as time intervals with sub-threshold activity across three most informative metrics (trunk, oral siphon opening width, and atrial siphon opening width).

To quantify behavioral dynamics in Ciona, we first identified three recurrent states: DPE, SePE, and StaPE, by aligning frame by frame annotations for three informative metrics. Short transient states (<2 s) were merged with adjacent states to reduce noise. From this cleaned sequence, state events were extracted per animal and condition, with start/end frames and durations calculated over a 15-min recording window. We computed inter event intervals, event durations and event frequencies (events/min) per state, and visualized state transitions using normalized chord diagrams. For each contraction event, we also quantified amplitude and the speeds of contraction and relaxation across morphometric metrics, after converting pixel measurements to micrometers.

To quantify how body shape changes across behavioral states, we extracted postures (shape outline) for each animal during DPE, SePE, and StaPE, states. From these outlines we calculated three shape metrics: ellipse aspect ratio which reflects body elongation and was estimated by fitting an ellipse to the body shape and taking the ratio of its long to short axes; solidity, which measures how tightly the body outline fits its convex hull and captures body curvature or folding; and body angle, defined as the orientation of the main body axis relative to the horizontal, indicating dorsal tilt. To account for differences in individual body size or orientation, each metric was normalized to each animal’s median across all three states. All metrics were averaged per individual and condition before plotting.

#### Extraction of unsupervised behavior features

We used the outline coordinates of each animal at each time point and converted them into closed polygons. Invalid shapes (e.g., incomplete or geometrically impossible contours) were excluded. We used Elliptic Fourier Descriptors (EFDs) to represent each outline as a set of harmonics that capture shape features at increasing detail. Fourier analysis was conducted using the pyefd package (https://github.com/hbldh/pyefd), following the Kuhl & Giardina (1982).^71^ Specifically, the elliptic_fourier_descriptors function was employed with a default order of 30, generating 120 coefficients (30 orders × 4 coefficients per order). This choice was confirmed visually by comparing original outlines with EFD reconstructions (error <1%). To ensure consistency, each shape was normalized for size and aligned to correct for left right mirroring. A data-driven scan across samples showed that 10 harmonics were sufficient to capture ≥99.9% of the overall shape information, and this value was used throughout for quantification.

We applied principal component analysis (PCA) to a 10-component amplitude spectrum to identify major modes of variation for static shape analysis. To include dynamics, we also computed how these amplitudes changed over time performing separate PCA.

These 10-dimensional amplitude vectors were then z-scored and submitted to Principal Component Analysis (PCA) using scikit-learn (Pedregosa et al., 2011).^117^ We retained the smallest number of PCs that explained ≥85% of the variance. The resulting PC1–PC5 scores therefore serve as our unsupervised “eigen shape” metrics or “eigen cionas”, capturing 85–90% of the observed outline variability. To verify interpretability, eigen shapes (principal components) were visualized and confirmed by reconstructing real contours associated with extreme PC scores for each animal and behavior state. Downstream analyses (state-classification, statistical comparisons) were performed on these five dimensions/“eigen cionas”.

For the shape space embedding and to visualize relationships between frames based on body shape, we used the first five principal components of shape (static PCs). To construct a shape-based behavioral space and visualize relationships between frames based on body posture, we first examined clustering structure in a balanced discovery subset of the embedded postural space (120,000 frames). Density-based clustering (HDBSCAN) was used to assess how the fraction of points classified as noise varied across parameter settings, allowing us to identify a stable clustering regime. Within this regime, Gaussian mixture models (GMMs) were compared using Akaike and Bayesian information criteria (AIC/BIC), leading to the selection of a 16-cluster solution. To identify stereotyped postural modes, each frame was clustered in this feature space using a GMM (K = 16). For visualization, the same features were embedded into two dimensions using UMAP (neighbors = 50, min_dist = 0.7), and cluster identities were overlaid on the embedding. Cluster labels inferred from the balanced discovery subset were subsequently propagated to the full dataset (∼980,000 frames) using k-nearest-neighbor (kNN) classification in feature space, yielding frame-wise postural-mode assignments for downstream analyses. Results were visualized by behavioral state and condition.

To model long-range postural dynamics, we applied hidden Markov modeling (HMM) to time-resolved postural features. Each frame was represented by the first five postural principal components (PC1–PC5), along with their temporal derivatives (dynamic PCs) to capture changes in posture over time. From these features, we additionally calculated postural speed and acceleration in principle component space. All features were computed independently for each individual and *Z* score normalized within animal to prevent individual-specific shift. The resulting feature set was used to train an eight-state Gaussian HMM, which was applied across all experimental conditions to infer frame-wise behavioral state sequences with 5 frames duration threshold. These inferred states were subsequently used to quantify behavioral organization through state occupancy and bout duration.

### Quantification and statistical analysis

The comparisons between states and conditions were performed using pairwise Mann Whitney U tests (ns *p* > 0.05; ∗*p* < 0.05; ∗∗*p* < 0.005; ∗∗∗*p* < 0.0001). For comparisons between line plots summarizing the probability of an adult Ciona being in one of the three states during the video recordings as a function of the stimulus used (no stimulus/control) we used Kruskal-Wallis test (∗*p* < 0.05).
