# Supplementary material for: Embodied postural dynamics underlie behavioral diversity in a benthic sessile chordate
Source: iScience. 2026 May 26;29(6):116104. doi: 10.1016/j.isci.2026.116104 (PMC13233770; doi:10.1016/j.isci.2026.116104)
Supplement: Document S1. Figures S1–S10 and Tables S1–S3 [file mmc1.pdf]

iScience, Volume *29*

Supplemental information

Embodied postural dynamics underlie behavioral diversity in a benthic sessile chordate

Oleg Tolstenkov, Sissel Norland, and Marios Chatzigeorgiou

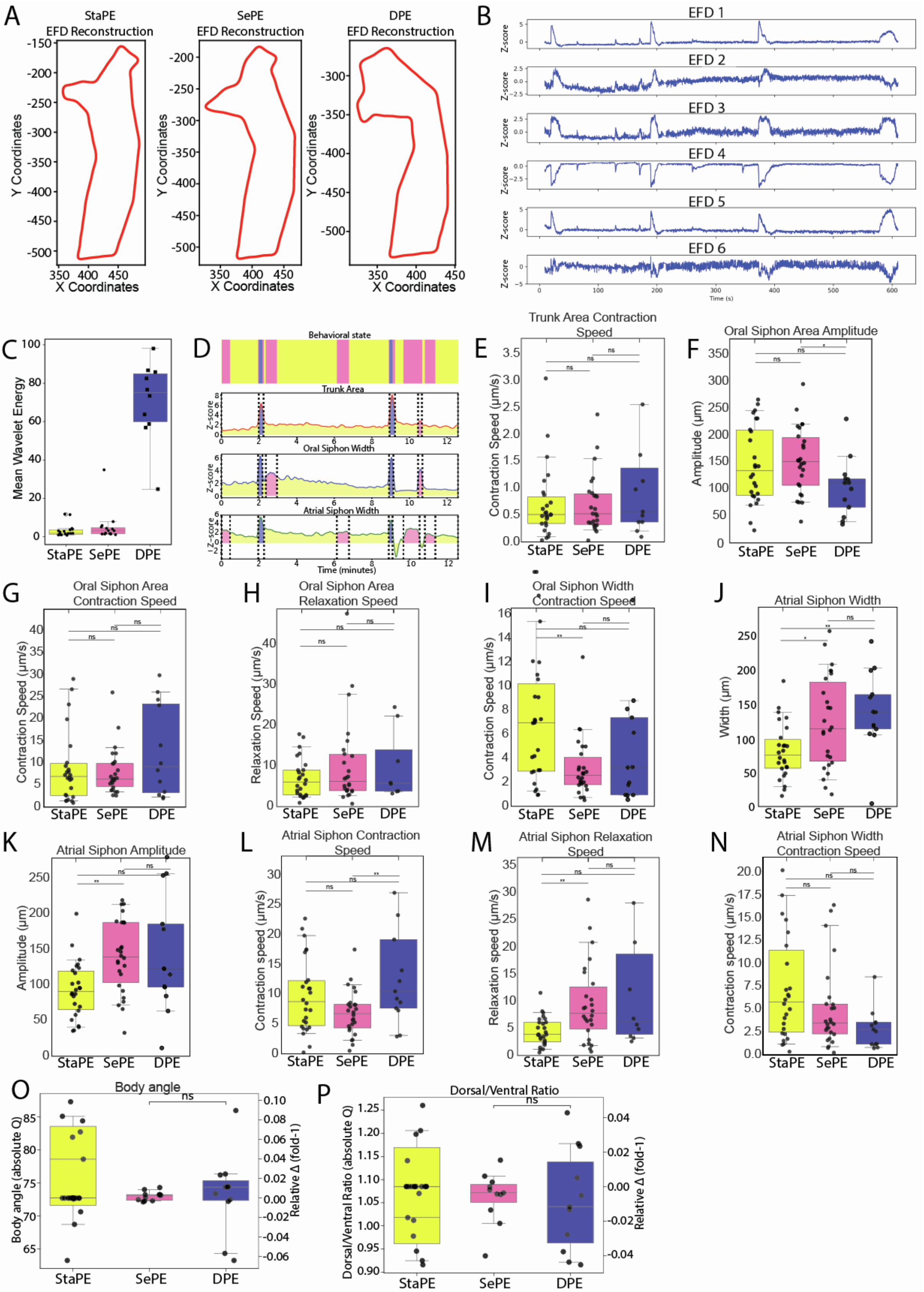
A
StaPE
EFD Reconstruction
SePE
EFD Reconstruction
DPE
EFD Reconstruction
Y Coordinates
X Coordinates
B
EFD 1
EFD 2
EFD 3
EFD 4
EFD 5
EFD 6
Z-score
Time (s)
C
Mean Wavelet Energy
StaPE SePE DPE
D
Behavioral state
Trunk Area
Oral Siphon Width
Atrial Siphon Width
Time (minutes)
E
Trunk Area Contraction Speed
Contraction Speed (μm/s)
StaPE SePE DPE
F
Oral Siphon Area Amplitude
Amplitude (μm)
StaPE SePE DPE
G
Oral Siphon Area Contraction Speed
Contraction Speed (μm/s)
StaPE SePE DPE
H
Oral Siphon Area Relaxation Speed
Relaxation Speed (μm/s)
StaPE SePE DPE
I
Oral Siphon Width Contraction Speed
Contraction Speed (μm/s)
StaPE SePE DPE
J
Atrial Siphon Width
Width (μm)
StaPE SePE DPE
K
Atrial Siphon Amplitude
Amplitude (μm)
StaPE SePE DPE
L
Atrial Siphon Contraction Speed
Contraction speed (μm/s)
StaPE SePE DPE
M
Atrial Siphon Relaxation Speed
Relaxation speed (μm/s)
StaPE SePE DPE
N
Atrial Siphon Width Contraction Speed
Contraction speed (μm/s)
StaPE SePE DPE
O
Body angle
Body angle (absolute Q)
Relative Δ (fold-1)
StaPE SePE DPE
P
Dorsal/Ventral Ratio
Dorsal/Ventral Ratio (absolute Q)
Relative Δ (fold-1)
StaPE SePE DPE

Figure S1. Parametrization of adult *Ciona* using interpretable features reveals three behavioral states (related to Figure 1)

(A) EFD reconstructions of the three different behavioral states of the adult animal used in Fig1C-D. (B) Representative example of the z-scored leading EFD predictors timeseries. (C) Mean wavelet energy of the EFD series for behavior states. (D) Ethogram for a representative *Ciona* adult from the control group (top panel) and the contraction signal for different body parts with mapped behavioral states (Blue = Dynamic postural engagement (DPE); Pink= Selective postural engagement (SePE) and yellow= Static postural engagement (StaPE). (E-P) Quantifications of body parts metrics and comparison between three behavioral states for control experiments. Mann Whitney U tests were used for statistical analysis (ns $p>0.05$; * $p<0.05$; ** $p<0.005$; *** $p<0.0001$). For all panels 43 animals were used.

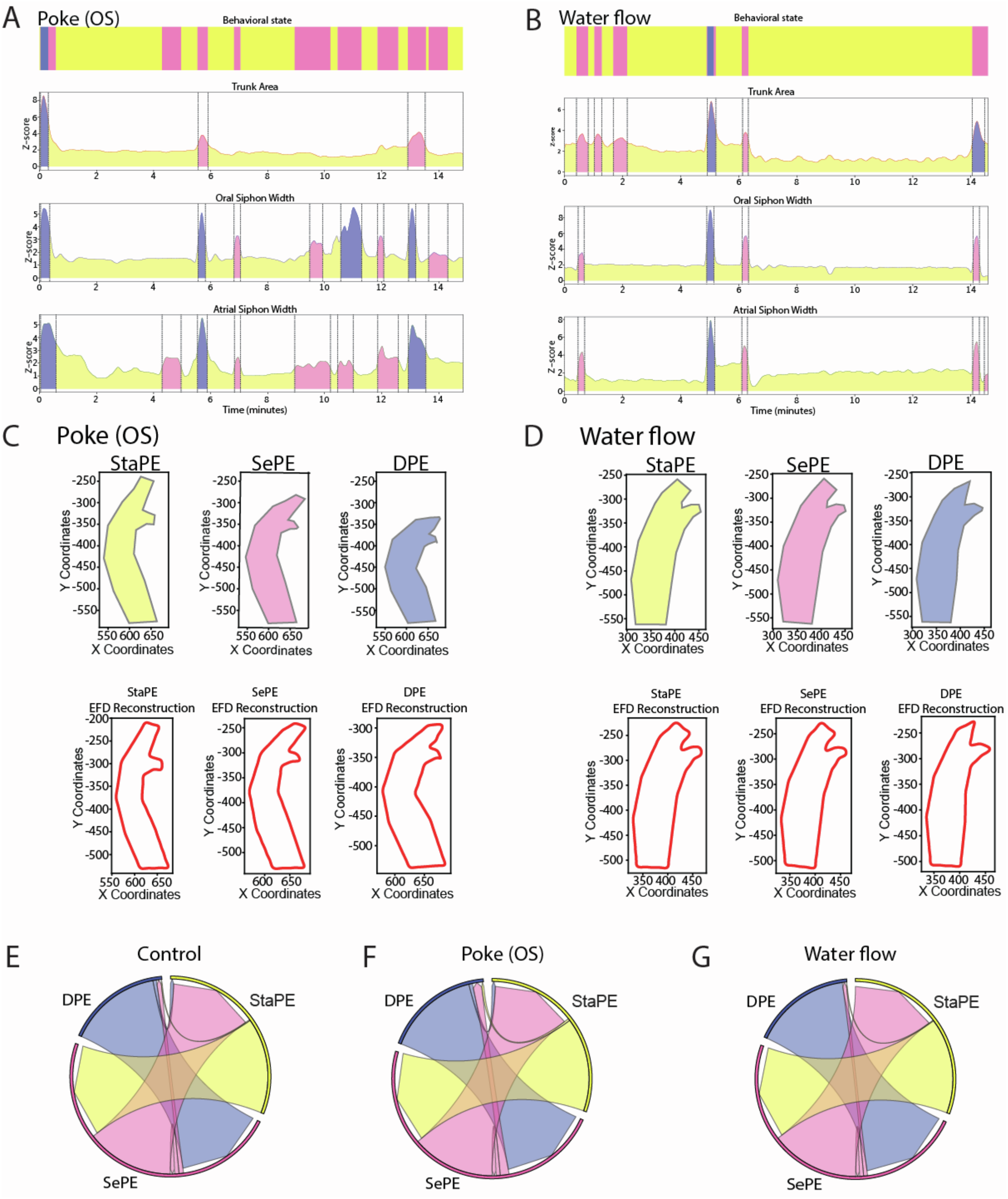

A Poke (OS)
Behavioral state
Trunk Area
Oral Siphon Width
Atrial Siphon Width
Z-score
Time (minutes)
B Water flow
C Poke (OS)
StaPE
SePE
DPE
X Coordinates
Y Coordinates
EFD Reconstruction
D Water flow
E Control
F Poke (OS)
G Water flow

Figure S2. Mechanical stimuli elicit distinct behavioral responses (related to Figure 2).

(A, B) Ethograms for a representative Ciona adult from the mechanical poke of the oral siphon (OS) group (A) and the water flow group (B). The contraction signals for different body parts with mapped behavioral states (Blue = Dynamic postural engagement (DPE); Pink= Selective postural engagement (SePE) and yellow= Static postural engagement (StaPE) are shown below each ethogram. (C, D) Reconstruction of *Ciona* contours from individual video frames that have been classified as belonging to one of the three states: Dynamic postural engagement (DPE); Selective postural engagement (SePE) and Static postural engagement (StaPE). Panels C and D correspond to the same animals as panels A and B, which were exposed to an OS poke stimulus and water flow stimuli respectively. (E-G) Chord diagrams showing the transitions between the three behavioral states in control unstimulated animals (E) or animals stimulated with an OS poke (F) or water flow cue (G). Number of animals, control n=52, poke OS=28 and water flow n=23.

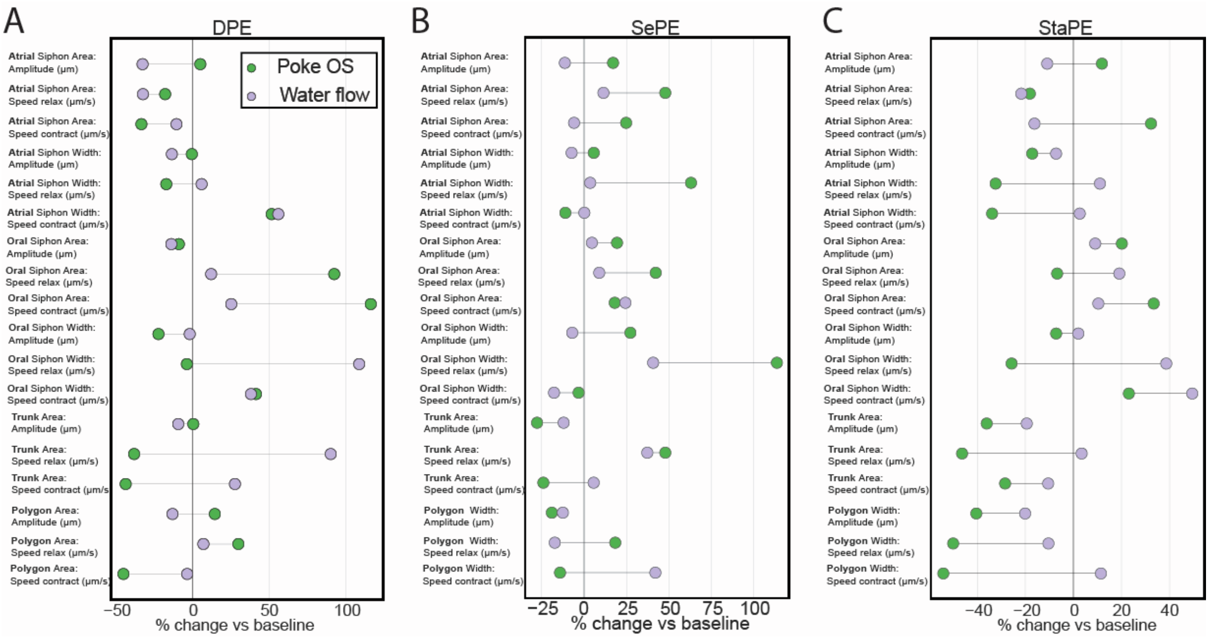

Figure S3. State-dependent modulation of postural features across stimulation conditions. (related to Figure 2).

Plots showing percent change relative to baseline=0 (control) for multiple postural and temporal features across behavioral states under oral siphon poke and water flow conditions. Each point represents the mean change across animals. Behavior states are dynamic postural engagement (A); selective postural engagement (B), and static postural engagement (C). Number of animals, control n=52, poke OS=28 and water flow n=23.

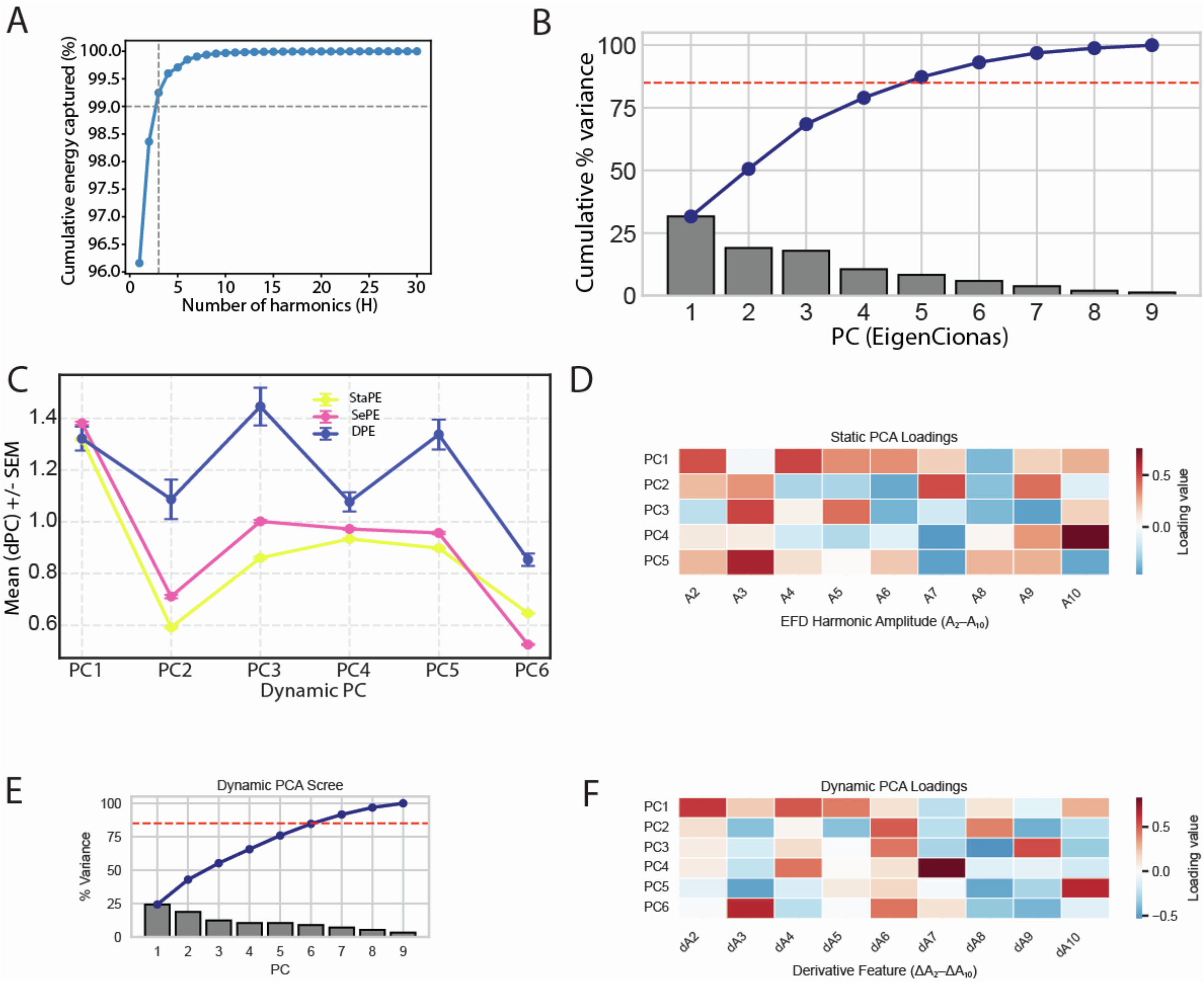

Figure S4. Five eigenciona shapes capture most of the postural variance of adult *Ciona* (related to Figure 3).

(A) EFD cumulative power curve showing the cumulative energy captured as a function of harmonics. Horizontal dashed line marks the 99% threshold of cumulative energy captured by 3 harmonics (vertical dashed line). (B) Five eigencionas are sufficient to explain 87% of the cumulative variance. Scree plot showing the explained variance by each eigenciona shapes. Red dotted line – threshold used for number of eigencionas selection (C) Plot quantifying the magnitude of dynamic eigen-shapes by behavior state. Error bars indicate S.E.M. (D) Dynamic PCA loading heatmap. (E) Eigenciona shapes PCA loading heatmap. Animals used for this analysis control =52; poke OS=28 and water flow =23.

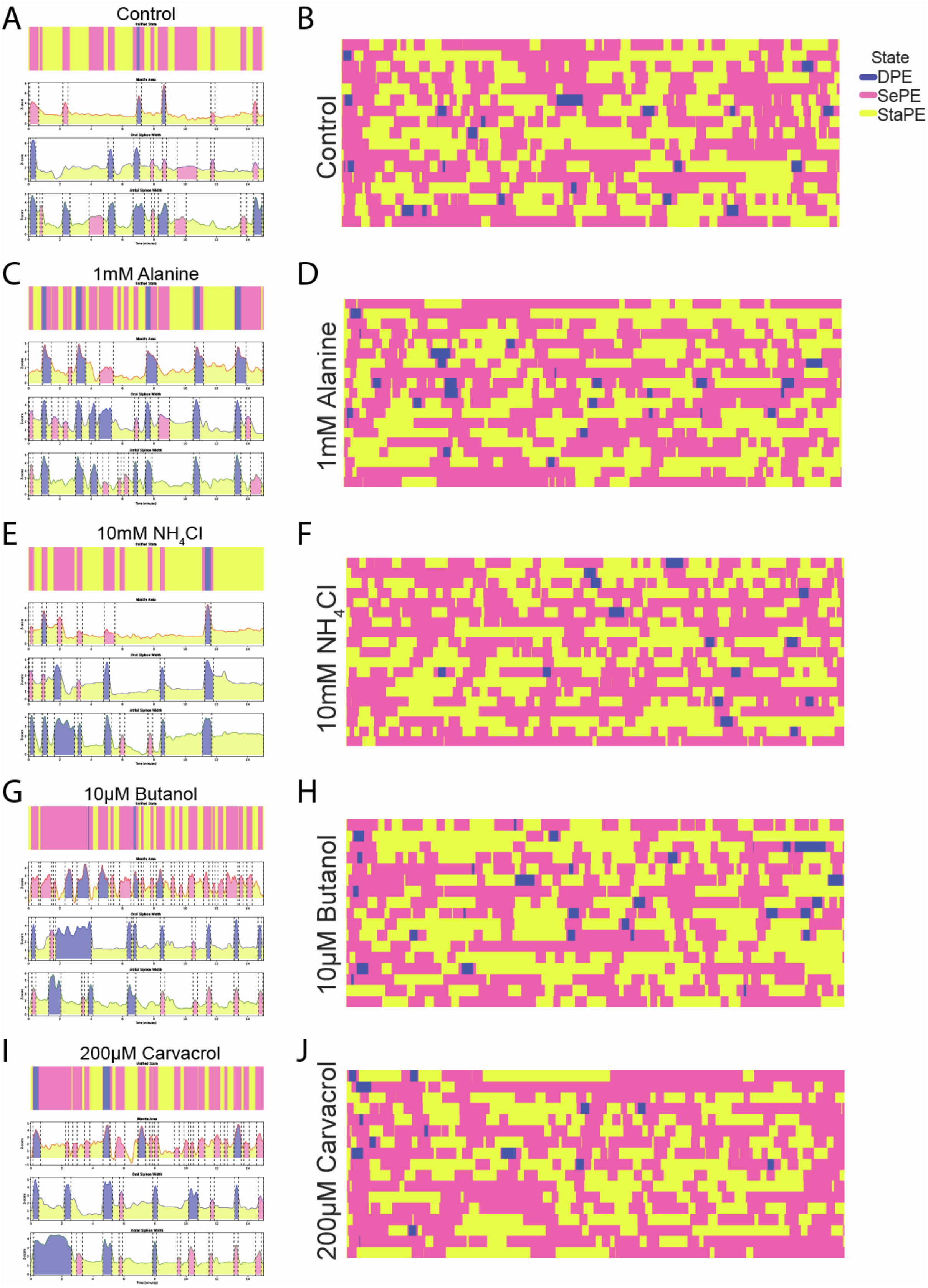

A
Control
B
Control
State
DPE
SePE
StaPE
C
1mM Alanine
D
1mM Alanine
E
10mM NH4Cl
F
10mM NH4Cl
G
10μM Butanol
H
10μM Butanol
I
200μM Carvacrol
J
200μM Carvacrol

Figure S5. Representative ethograms and contraction dynamics across chemosensory stimuli (related to Figure 5).

(A, C, E, G, I) Representative ethograms from adult *Ciona* under different chemosensory stimuli, showing behavioral state sequences over time. For each condition, the top panel shows the ethogram of a representative individual, while traces below display contraction dynamics of informative body regions feature with behavioral states overlaid. Behavioral states are color-coded as Dynamic Postural Engagement (DPE; blue), Selective Postural Engagement (SePE; pink), and Static Postural Engagement (StaPE; yellow). (B, D, F, H, J) Ethogram heatmaps for pooled individuals. Panels correspond to the following conditions: control (A–B), 1 mM alanine (C–D), 10 mM NH_4Cl (E–F), 10 μM butanol (G–H), and 200 μM carvacrol (I–J). Sample sizes (number of animals): control (n = 17), alanine (1 mM, n = 19), NH_4Cl (10 mM, n = 19), butanol (10 μM, n = 17), and carvacrol (200 μM, n = 17).

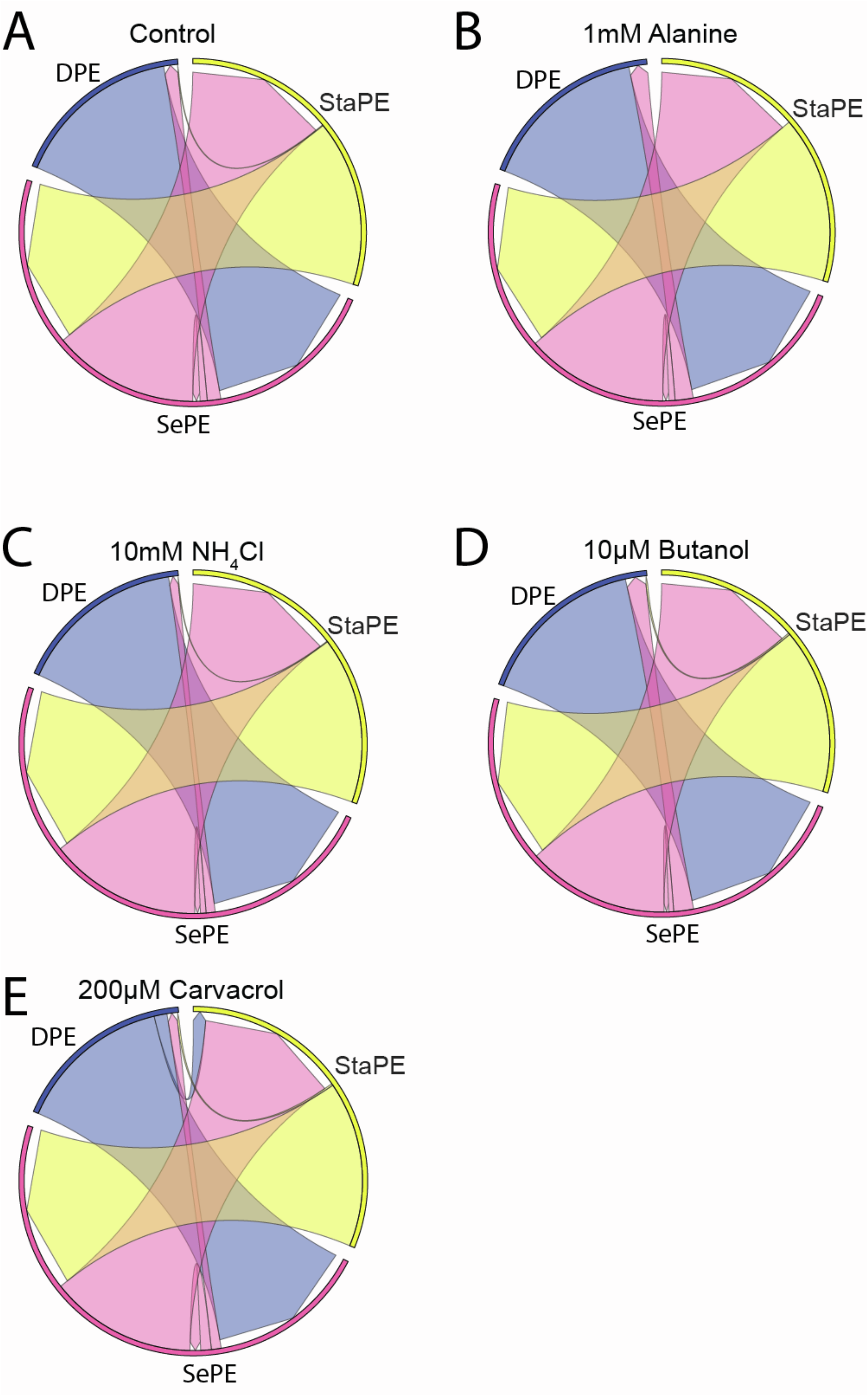
A
Control
DPE
StaPE
SePE
B
1mM Alanine
DPE
StaPE
SePE
C
10mM NH_4Cl
DPE
StaPE
SePE
D
10μM Butanol
DPE
StaPE
SePE
E
200μM Carvacrol
DPE
StaPE
SePE

Figure S6. Transition probabilities between behavioral states in response to chemosensory stimuli (related to Figure 5).

(A-E) Chord diagrams showing the transitions between the three behavioral states in control (A) 1 mM alanine (B), 10 mM NH_4Cl (C), 10 μM butanol (D), and 200 μM carvacrol (E). Sample sizes (number of animals): control (n = 17), alanine (1 mM, n = 19), NH_4Cl (10 mM, n = 19), butanol (10 μM, n = 17), and carvacrol (200 μM, n = 17).

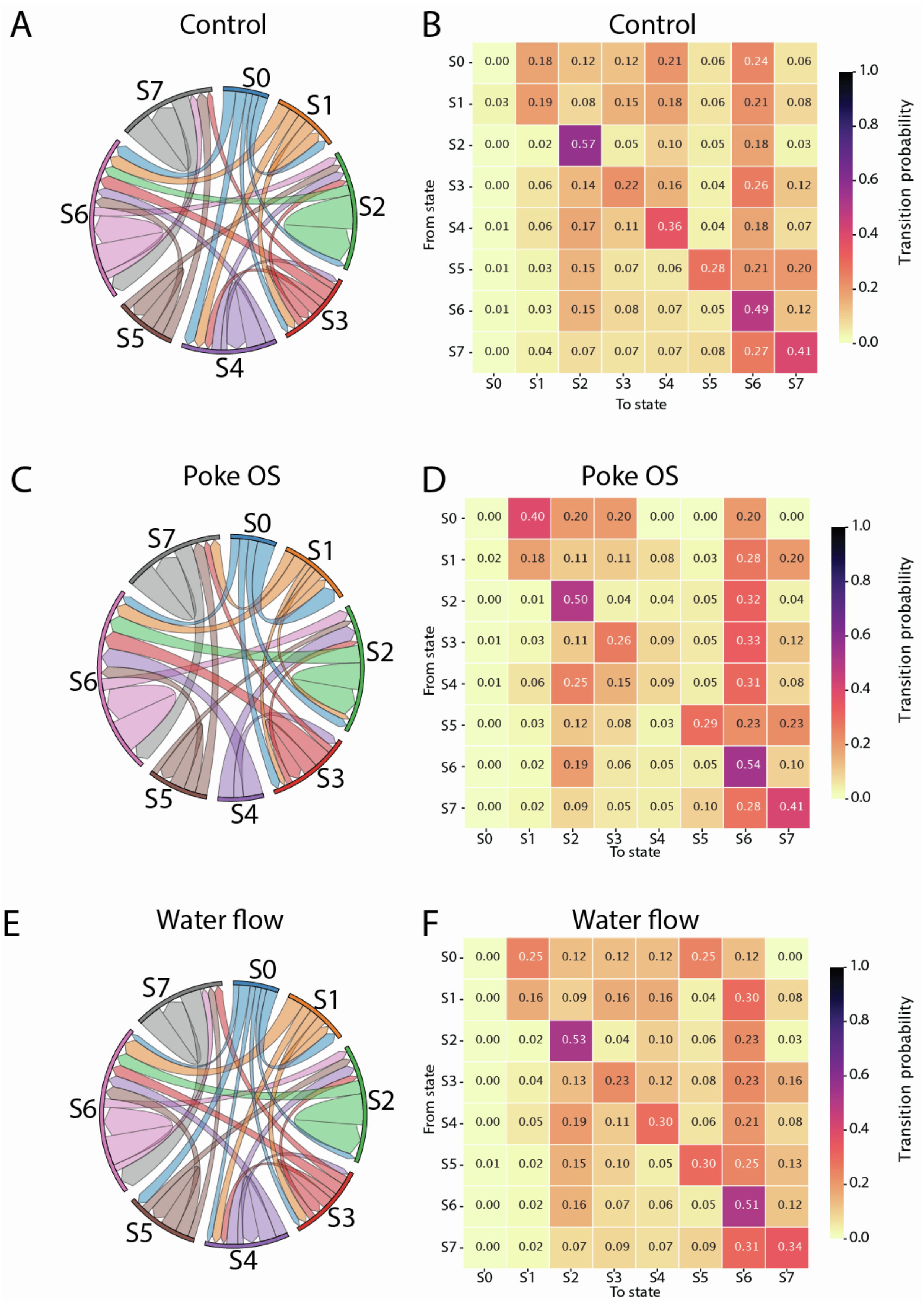

A
Control
S0
S1
S2
S3
S4
S5
S6
S7
B
Control
From state
To state
S0 0.00 0.18 0.12 0.12 0.21 0.06 0.24 0.06
S1 0.03 0.19 0.08 0.15 0.18 0.06 0.21 0.08
S2 0.00 0.02 0.57 0.05 0.10 0.05 0.18 0.03
S3 0.00 0.06 0.14 0.22 0.16 0.04 0.26 0.12
S4 0.01 0.06 0.17 0.11 0.36 0.04 0.18 0.07
S5 0.01 0.03 0.15 0.07 0.06 0.28 0.21 0.20
S6 0.01 0.03 0.15 0.08 0.07 0.05 0.49 0.12
S7 0.00 0.04 0.07 0.07 0.07 0.08 0.27 0.41
S0 S1 S2 S3 S4 S5 S6 S7
Transition probability
1.0
0.8
0.6
0.4
0.2
0.0
C
Poke OS
S0
S1
S2
S3
S4
S5
S6
S7
D
Poke OS
From state
To state
S0 0.00 0.40 0.20 0.20 0.00 0.00 0.20 0.00
S1 0.02 0.18 0.11 0.11 0.08 0.03 0.28 0.20
S2 0.00 0.01 0.50 0.04 0.04 0.05 0.32 0.04
S3 0.01 0.03 0.11 0.26 0.09 0.05 0.33 0.12
S4 0.01 0.06 0.25 0.15 0.09 0.05 0.31 0.08
S5 0.00 0.03 0.12 0.08 0.03 0.29 0.23 0.23
S6 0.00 0.02 0.19 0.06 0.05 0.05 0.54 0.10
S7 0.00 0.02 0.09 0.05 0.05 0.10 0.28 0.41
S0 S1 S2 S3 S4 S5 S6 S7
Transition probability
1.0
0.8
0.6
0.4
0.2
0.0
E
Water flow
S0
S1
S2
S3
S4
S5
S6
S7
F
Water flow
From state
To state
S0 0.00 0.25 0.12 0.12 0.12 0.25 0.12 0.00
S1 0.00 0.16 0.09 0.16 0.16 0.04 0.30 0.08
S2 0.00 0.02 0.53 0.04 0.10 0.06 0.23 0.03
S3 0.00 0.04 0.13 0.23 0.12 0.08 0.23 0.16
S4 0.00 0.05 0.19 0.11 0.30 0.06 0.21 0.08
S5 0.01 0.02 0.15 0.10 0.05 0.30 0.25 0.13
S6 0.00 0.02 0.16 0.07 0.06 0.05 0.51 0.12
S7 0.00 0.02 0.07 0.09 0.07 0.09 0.31 0.34
S0 S1 S2 S3 S4 S5 S6 S7
Transition probability
1.0
0.8
0.6
0.4
0.2
0.0

Figure S7. Behavioral state transition structure under global mechanical stimulation (related to Figure 6).

(A, C, E) Chord plots showing behavioral state transition probabilities inferred from the HMM under control (A), oral siphon poke (C), and water flow (E) conditions. Only transitions exceeding 10% probability are shown for clarity. (B, D, F) Corresponding full transition probability matrices for control (B), oral siphon poke (D), and water flow (F) conditions. Sample sizes: control n=52, poke OS n=28 and water flow n=23.

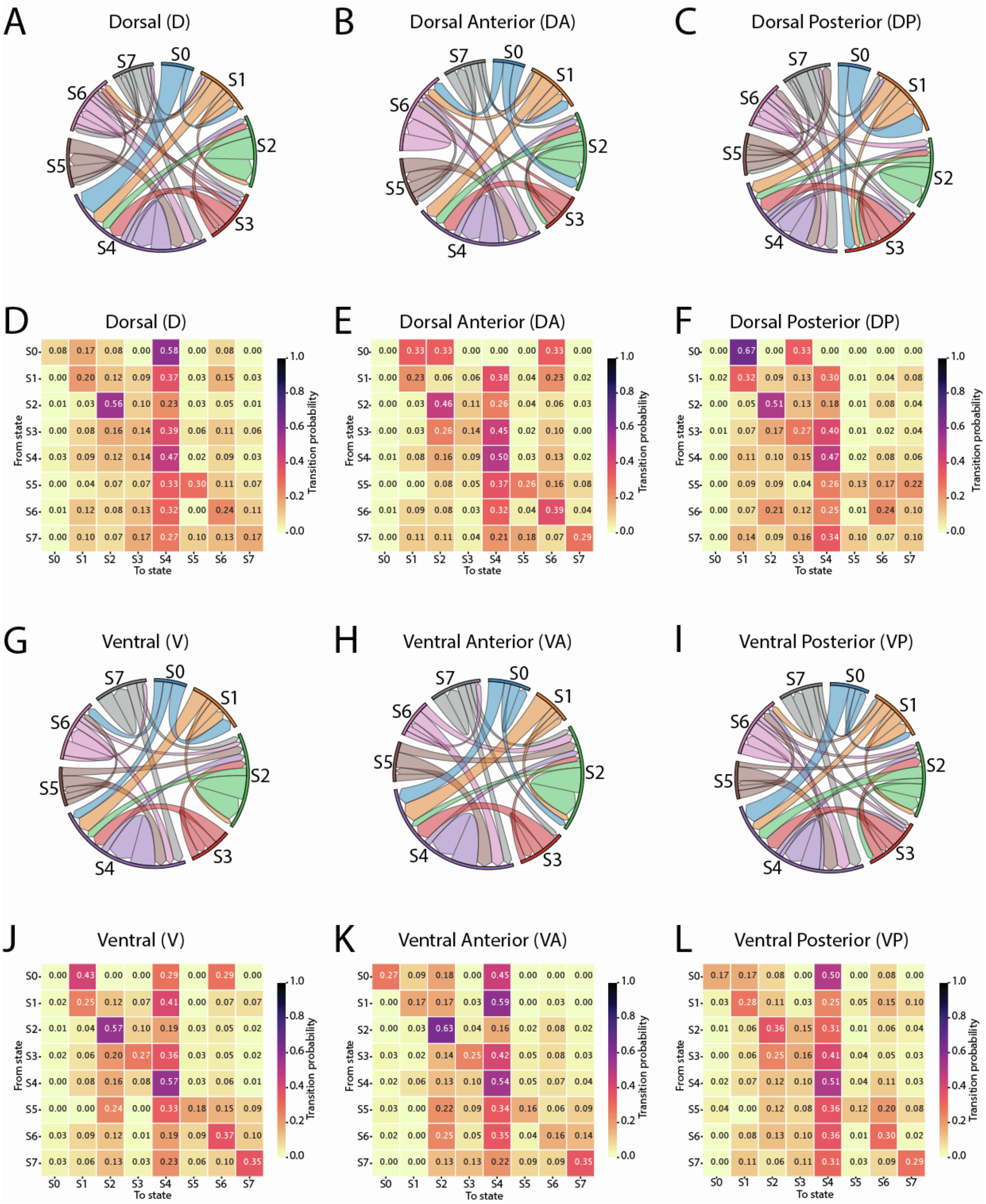
A Dorsal (D)
B Dorsal Anterior (DA)
C Dorsal Posterior (DP)
S0 S1 S2 S3 S4 S5 S6 S7
D Dorsal (D)
S0 0.08 0.17 0.08 0.00 0.58 0.00 0.08 0.00
S1 0.00 0.20 0.12 0.09 0.37 0.03 0.15 0.03
S2 0.01 0.03 0.56 0.10 0.23 0.03 0.05 0.01
S3 0.00 0.08 0.16 0.14 0.39 0.06 0.11 0.06
S4 0.03 0.09 0.12 0.14 0.47 0.02 0.09 0.03
S5 0.00 0.04 0.07 0.07 0.33 0.30 0.11 0.07
S6 0.01 0.12 0.08 0.13 0.32 0.00 0.24 0.11
S7 0.00 0.10 0.07 0.17 0.27 0.10 0.13 0.17
E Dorsal Anterior (DA)
S0 0.00 0.33 0.33 0.00 0.00 0.00 0.33 0.00
S1 0.00 0.23 0.06 0.06 0.38 0.04 0.23 0.02
S2 0.00 0.03 0.46 0.11 0.26 0.04 0.06 0.03
S3 0.00 0.03 0.26 0.14 0.45 0.02 0.10 0.00
S4 0.01 0.08 0.16 0.09 0.50 0.03 0.13 0.02
S5 0.00 0.00 0.08 0.05 0.37 0.26 0.16 0.08
S6 0.01 0.09 0.08 0.03 0.32 0.04 0.39 0.04
S7 0.00 0.11 0.11 0.04 0.21 0.18 0.07 0.29
F Dorsal Posterior (DP)
S0 0.00 0.67 0.00 0.33 0.00 0.00 0.00 0.00
S1 0.02 0.32 0.09 0.13 0.30 0.01 0.04 0.08
S2 0.00 0.05 0.51 0.13 0.18 0.01 0.08 0.04
S3 0.01 0.07 0.17 0.27 0.40 0.01 0.02 0.04
S4 0.00 0.11 0.10 0.15 0.47 0.02 0.08 0.06
S5 0.00 0.09 0.09 0.04 0.26 0.13 0.17 0.22
S6 0.00 0.07 0.21 0.12 0.25 0.01 0.24 0.10
S7 0.00 0.14 0.09 0.16 0.34 0.10 0.07 0.10
From state
To state
Transition probability
0.0 0.2 0.4 0.6 0.8 1.0
G Ventral (V)
H Ventral Anterior (VA)
I Ventral Posterior (VP)
J Ventral (V)
S0 0.00 0.43 0.00 0.00 0.29 0.00 0.29 0.00
S1 0.02 0.25 0.12 0.07 0.41 0.00 0.07 0.07
S2 0.01 0.04 0.57 0.10 0.19 0.03 0.05 0.02
S3 0.02 0.06 0.20 0.27 0.36 0.03 0.05 0.02
S4 0.00 0.08 0.16 0.08 0.57 0.03 0.06 0.01
S5 0.00 0.00 0.24 0.00 0.33 0.18 0.15 0.09
S6 0.03 0.09 0.12 0.01 0.19 0.09 0.37 0.10
S7 0.03 0.06 0.13 0.03 0.23 0.06 0.10 0.35
K Ventral Anterior (VA)
S0 0.27 0.09 0.18 0.00 0.45 0.00 0.00 0.00
S1 0.00 0.17 0.17 0.03 0.59 0.00 0.03 0.00
S2 0.00 0.03 0.63 0.04 0.16 0.02 0.08 0.02
S3 0.03 0.02 0.14 0.25 0.42 0.05 0.08 0.03
S4 0.02 0.06 0.13 0.10 0.54 0.05 0.07 0.04
S5 0.03 0.00 0.22 0.09 0.34 0.16 0.06 0.09
S6 0.02 0.00 0.25 0.05 0.35 0.04 0.16 0.14
S7 0.00 0.00 0.13 0.13 0.22 0.09 0.09 0.35
L Ventral Posterior (VP)
S0 0.17 0.17 0.08 0.00 0.50 0.00 0.08 0.00
S1 0.03 0.28 0.11 0.03 0.25 0.05 0.15 0.10
S2 0.01 0.06 0.36 0.15 0.31 0.01 0.06 0.04
S3 0.00 0.06 0.25 0.16 0.41 0.04 0.05 0.03
S4 0.02 0.07 0.12 0.10 0.51 0.04 0.11 0.03
S5 0.04 0.00 0.12 0.08 0.36 0.12 0.20 0.08
S6 0.00 0.08 0.13 0.10 0.36 0.01 0.30 0.02
S7 0.00 0.11 0.06 0.11 0.31 0.03 0.09 0.29

Figure S8. Region-specific mechanical stimulation modulates behavioral state transitions (related to Figure 6).

(A, C, E, G, I, K) Chord plots showing behavioral state transitions following poke stimulation at different body regions: dorsal (A), dorsal anterior (C), dorsal posterior (E), ventral (G), ventral anterior (I), and ventral posterior (K). Transitions shown exceed a 10% probability threshold. (B, D, F, H, J, L) Corresponding full transition probability matrices for each stimulation site. Sample sizes per condition: OS n=28, D n=7, V n=8, DA n=8, DP n=10, VA n=6, VP n=7.

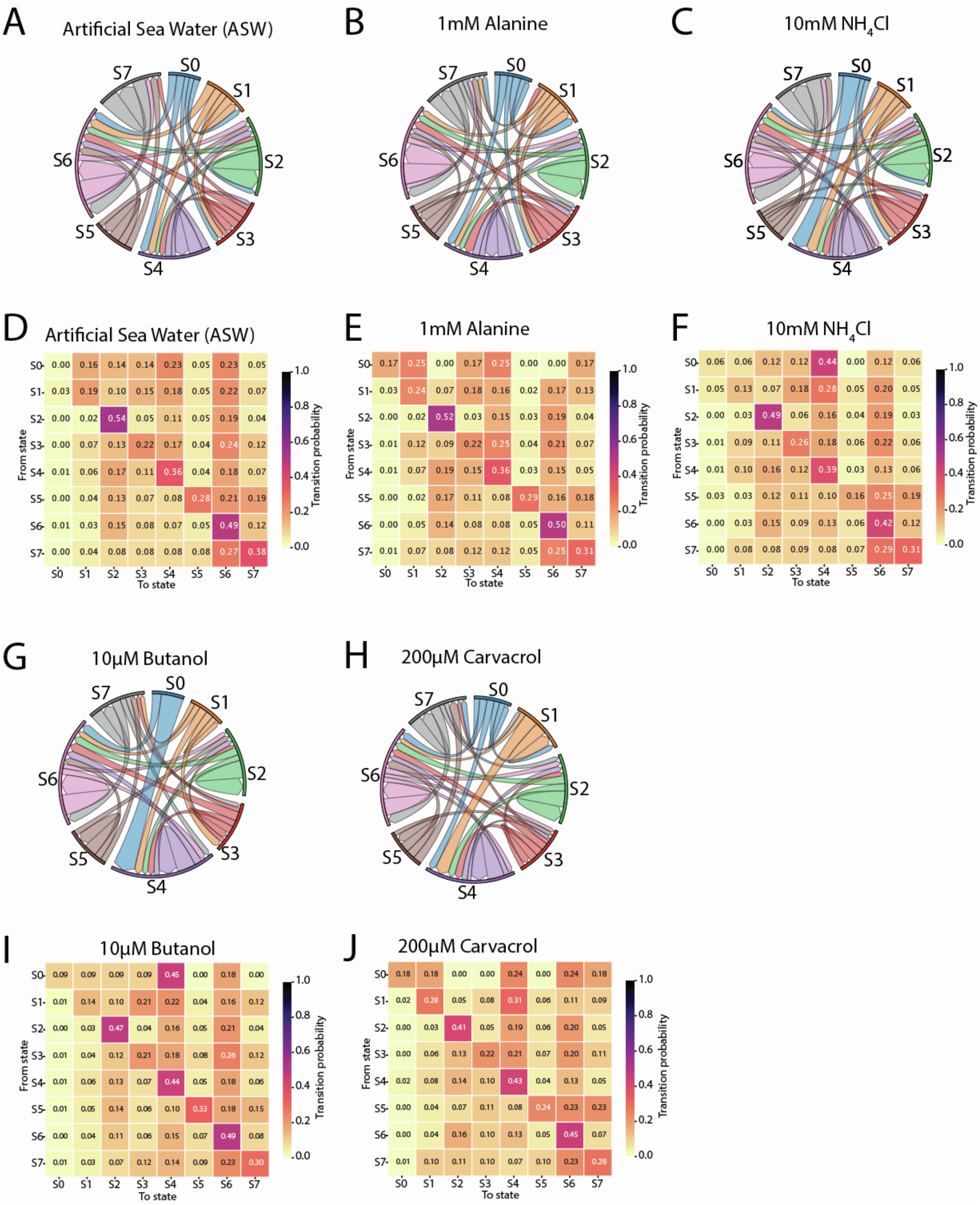
A
Artificial Sea Water (ASW)
B
1mM Alanine
C
10mM NH4Cl
S0
S1
S2
S3
S4
S5
S6
S7
D
Artificial Sea Water (ASW)
E
1mM Alanine
F
10mM NH4Cl
From state
To state
Transition probability
G
10μM Butanol
H
200μM Carvacrol
I
10μM Butanol
J
200μM Carvacrol

Figure S9. Chemosensory cues differentially bias behavioral state transitions (related to Figure 6).

(A, C, E, G, I) Chord plots showing behavioral state transition probabilities under artificial seawater control (A), alanine (C), ammonia (E), butanol (G), and carvacrol (I) stimuli. Only transitions exceeding 10% probability are displayed. (B, D, F, H, J) Corresponding full transition probability matrices for each chemosensory condition.). Sample sizes per condition: Control n=17, 1 mM alanine n=19, 10 mM NH_4Cl n=19, 10 μM butanol n=17, and 200 μM Carvacrol n=17.

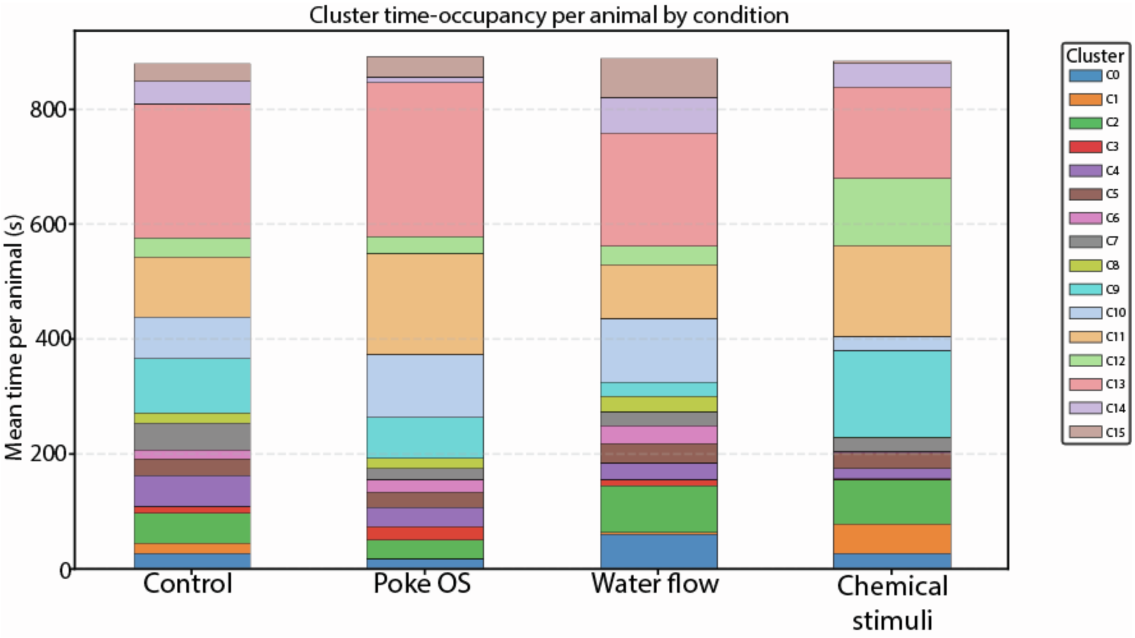

Figure S10 Mean time occupancy of postural clusters per animal across experimental conditions, shown as a stacked bar plot (related to Figure 7). Sample sizes used control (n=52), oral siphon poke (n=28), water flow (n=23) and pooled chemical stimuli conditions (n=89 in total).

Table S1 Description of behavioral states, eigencionas and clusters based on anatomical features. Related to Figures 1-7.

	Atrial siphon	Oral siphon	Trunk	Dorsal side	Ventral Side
DPE	The atrial siphon remains open, with the highest closing and relaxation speeds during both closing and opening, and the highest amplitude.	The oral siphon remains open with maximum aperture and any small closure of the aperture returns to previous size with a high speed	The trunk is highly contracted, with the highest trunk area change amplitude and the fastest relaxation speed.	Curved with the maximum deformation.	Curved with the maximum deformation.
SePE	The atrial siphon can be closed or partially open, with the medium closing and relaxation speeds and amplitude.	The oral siphon aperture is partially closed and returns to full aperture with a medium speed	The trunk is partially contracted, with the medium change amplitude and speed.	Curved with the medium deformation.	Curved with the medium deformation.
StaPE	The atrial siphon is partially closed, with the lowest closing and relaxation speeds and the lowest amplitude.	The oral siphon aperture is the narrowest amongst the three behavioral states and returns to full aperture with a low speed	Trunk is upright (not contracted), with the lowest change amplitude and speed	Concave with minimal deformation.	Concave with minimal deformation.
EC1	The atrial siphon fully expanded in length, aperture is open	The oral siphon is fully expanded in length, aperture is open	Trunk is upright (not contracted)	Concave	Concave
EC2	The atrial siphon is contracted and aperture is closed	The oral siphon is contracted and aperture is closed	Trunk is contracted	Curved, Convex	Curved, Convex
EC3	The atrial siphon is contracted and aperture is closed	The oral siphon is contracted and aperture is closed	Trunk is upright (not contracted)	Concave	Concave
EC4	The atrial siphon is contracted and aperture is closed	The oral siphon is contracted and aperture is closed	Trunk is contracted	Curved, Convex	Curved, Convex
EC5	The atrial siphon is not contracted and aperture is open	The oral siphon is not contracted and aperture is open	Trunk is upright (not contracted)	Concave	Concave
HMM State 0	The atrial siphon aperture is open and fully expanded in length	The oral siphon aperture is open, and fully expanded in length.	Trunk is upright (not contracted)	Concave	Concave
HMM State 1	The atrial siphon is open, and partially contracted in length	The oral siphon is fully open and extended	Trunk is upright (not contracted)	Concave	Concave
HMM State 2	The atrial siphon is closed, and fully contracted in length	The oral siphon is fully open and extended	Trunk is contracted	Curved, Convex	Curved, Convex
HMM State 3	The atrial siphon is partially extended and fully contracted in length	The oral siphon is fully open and extended	Trunk is contracted	Curved, Convex	Curved, Convex
HMM State 4	The atrial siphon is fully extneded but with partially closed aperture	The oral siphon is fully extneded but with partially closed aperture	Trunk is thinner than control	Concave	Concave
HMM State 5	The atrial siphon is fully contracted and fully closed	The oral siphon is fully extended and fully open aperture	Trunk is contracted	Curved, Convex	Curved, Convex
HMM State 6	The atrial siphon area is reduced but not fully contracted but the aparture is closed	The oral siphon is partially contracted but the aperture is fully closed.	Trunk is partially contracted	Concave	Concave

HMM State 7	The atrial siphon is fully contracted and the apparture fully closed	The oral siphon is partially contracted but the aperture is fully open.	Trunk is contracted	Curved, Convex	Curved, Convex
UMAP Cluster 0	The atrial siphon is contracted and aperture is closed	The oral siphon is extended and partially closed aperture.	Trunk is contracted	Curved, Convex	Curved, Convex
UMAP Cluster 1	The atrial siphon area is reduced and partially contracted, aperture is partially closed	The oral siphon is extended with fully open aperture.	Trunk partially contracted	Curved, Concave	Curved, Concave
UMAP Cluster 2	The atrial siphon area is reduced but not fully contracted but the aparture is closed	The oral siphon is extended with fully open aperture.	Trunk is partially contracted	Partially curved, concave	Partially curved, concave
UMAP Cluster 3	The atrial siphon area is reduced but not fully contracted but the aparture is closed	The oral siphon is extended and partially closed aperture.	Trunk is partially contracted	Concave	Concave
UMAP Cluster 4	The atrial siphon area is reduced but not fully contracted but the aparture is closed	The oral siphon is contracted but with fully open aperture.	Trunk is partially contracted	Partially curved, concave	Partially curved, concave
UMAP Cluster 5	The atrial siphon is contracted and aperture is partially closed	The oral siphon aperture is open, and fully expanded in length.	Trunk is upright (not contracted)	Concave	Concave
UMAP Cluster 6	The atrial siphon aperture is open and fully expanded in length	The oral siphon aperture is open, and fully expanded in length.	Trunk is upright (not contracted)	Concave	Concave
UMAP Cluster 7	The atrial siphon is contracted and aperture is partially closed	The oral siphon aperture is open, and fully expanded in length.	Trunk partially contracted	Curved, Concave	Curved, Concave
UMAP Cluster 8	The atrial siphon fully expanded in length, aperture is open	The oral siphon is fully expanded in length, aperture is open	Trunk is upright (not contracted)	Concave	Concave
UMAP Cluster 9	The atrial siphon is contracted and aperture is partially closed	The oral siphon is fully expanded in length, aperture is open	Trunk is contracted	Curved, Convex	Curved, Convex
UMAP Cluster 10	The atrial siphon fully expanded in length, aperture is open	The oral siphon is partially contracted but the aperture is fully closed.	Trunk is contracted	Curved, Convex	Curved, Convex
UMAP Cluster 11	The atrial siphon fully expanded in length, aperture is closed	The oral siphon is extended and partially closed aperture.	Trunk is upright (not contracted)	Concave	Concave
UMAP Cluster 12	The atrial siphon area is reduced but not fully contracted but the aparture is closed	The oral siphon is extended and partially closed aperture.	Trunk is upright	Concave	Concave
UMAP Cluster 13	The atrial siphon is partially contracted and aperture is partially closed	The oral siphon is extended and partially closed aperture.	Trunk is upright	Concave	Concave
UMAP Cluster 14	The atrial siphon fully expanded in length, aperture is closed	The oral siphon is extended and partially closed aperture.	Trunk is upright (not contracted)	Concave	Concave
UMAP Cluster 15	The atrial siphon is contracted and aperture is partially closed	The oral siphon aperture is open, and fully expanded in length.	Trunk partially contracted	Curved, Concave	Curved, Concave

Table S2 Description of behavioral responses of adult *Cionas* to different mechanical and chemical stimuli. Related to Figures 2-7.

	Atrial siphon	Oral siphon	Trunk
Poke OS	Increased relaxation speed; participates in closure/withdrawal patterns.	Strong increase in area & width, increase in relaxation speed, often closes during defensive response.	Modest changes compared to siphons; full-body contraction events are associated with DPE events.
Water flow	The partial closure and withdrawal of the siphon is common.	The oral siphon width is strongly modulated. There is an increase in contraction and relaxation dynamics. The partial withdrawal of the oral siphon without a simultaneous contraction of the trunk is common	No immediate full-body contraction, but there is an incrase in the relaxation speed following contraction events when they happen.
Poke VA	Relative decrease in atrial siphon area and width relative to OS poking. Only VA stimulation leads to a small increase in atrial siphon relaxation speed relative to OS stimulation.	No significant change in terms of oral siphon dynamics relative to OS poking. Decrease in amplitude of oral siphon width and area relative to OS poking.	The contraction and relaxation of the trunk (and the entire body as a whole) is faster compared to when the OS is stimulated. VA shows the largest increase in relaxation of the trunk amongst all sites and it is also amongst the fastest in terms of contraction speed (again relative to OS).
Poke V	P stimulation leads to Reduced opening amplitudes. Specifically a decease in atrial siphon area and width relative to poke OS (i.e more contracted atrial siphon).	No significant change in terms of oral siphon dynamics relative to OS poking. Decrease in amplitude of oral siphon width and area relative to OS poking.	The contraction and relaxation of the trunk (and the entire body as a whole) is faster compared to when the OS is stimulated.
Poke VP	VP stimulation leads to the largest decrease atrial siphon area and width relative to OS poke stimulation. VP stimulation leads to a decrease in atrial siphon area relaxation speed.	No significant change in terms of oral siphon dynamics relative to OS poking. Decrease in amplitude of oral siphon width and area relative to OS poking.	The contraction and relaxation of the trunk (and the entire body as a whole) is faster compared to when the OS is stimulated.
Poke DA	DA stimulation leads to the smallest decrease in atrial siphon area and width amongst the different stimuatoin sites (relative to OS poke). Contractin speed of the atrial siphon area is slower than poke OS.	Reduced opening amplitude vs OS.	Increased trunk amplitude and contraction speed, though less than V/VA/VP.
Poke D	Amplitude (opening) reduced vs OS poke.	Amplitude (opening) reduced vs OS poke.	Increased trunk area amplitudes; increased contraction speeds.
Poke DP	Reduced opening amplitudes. Specifically, decrease in atrial siphon area and width relative to OS poking (i.e. more contracted atrial siphon). The contraction and relaxation of the kinematics showed a similar behavior to OS poking	No significant change in terms of oral siphon dynamics relative to OS poking. Decrease in amplitude of oral siphon width and area relative to OS poking.	Increased amplitudes; highest whole-body relaxation speed.
10mM NH4Cl	the atrial siphon expands after stimulus presentation	Strong increase in oral siphon dimensions (Width) and modest increase in the speed with which the oral siphon relaxes to bring about this increase in width	The trunk area increases after stimulus presentation
1mM Alanine	no change in dimensions and speed of movement	no change in oral siphon dimensions and modest increase in the speed with which the oral siphon width relaxes	The trunk area increases after stimulus presentation
10uM Butanol	no change in dimensions and very modest increase in relaxation and contraction speed of the atrial siphon	no change in oral siphon dimensions and big increase in the speed with which the oral siphon width relaxes	The trunk area increases after stimulus presentation. This the largest change amongst the chemical stimuli
200uM Cavacrol	the atrial siphon expands after stimulus presentation with the highest increase in relaxation speed relative to the other stimuli	no change in oral siphon dimensions and modest increase in the speed with which the oral siphon width relaxes	increase in contraction amplitude after stimulation.

Footnotes for Table S2

Across all conditions, siphons are the primary effectors (largest, fastest stimulus-driven changes), while the trunk/whole-body often shows secondary modulation—strongest with VA/V pokes and certain chemicals (butanol, carvacrol).

OS poke is unique in eliciting immediate, strong defensive response with full contraction and a subsequent bias toward a closure of siphons, whereas water flow drives delayed contraction engagement with siphon-centric withdrawal.

Regional pokes (D/DA/DP/V/VA/VP) systematically reduce siphon opening yet increase trunk/whole-body amplitudes and speeds, with site-specific peaks (e.g., V for contraction speed, VA for expansion, DP for relaxation).

Chemical cues are compound-specific: NH_4Cl maximizes siphon width amplitude; butanol maximizes whole-body/trunk amplitude with fast siphon kinetics; carvacrol induces the broadest, longest-lasting state changes

Table S3 Parameters of mechanical stimuli delivered to *Ciona* adults. Relevant to Figures 2, 3, 4, 6 and 7.

		OS			VA			V	
Animal	Angle (°)	Stim. Duration (ms)	Indentation (mm)	Angle (°)	Stim. Duration (ms)	Indentation (mm)	Angle (°)	Stim. Duration (ms)	Indentation (mm)
1	6,2	750	2.9	36,5	1000	5.4	42,2	800	7.8
2	5,5	850	2.7	39,6	1100	3.7	37,1	950	9.1
3	8,2	800	3.0	45,1	800	3.0	38,0	850	5.9
4	9,8	1000	2.5	47,8	850	5.7	41,6	1000	4.4
5	9,3	750	3.6	42,0	950	7.2	34,3	900	3.3
6	8,2	900	3.1	41,2	1050	4.0	35,1	1050	4.6
7	14,5	1050	4.0				48,3	750	6.9
8	23,4	1100	4.3				44,1	1000	7.4
9	16,3	1000	3.5						
10	17,0	950	3.2						
11	17,7	900	4.1						
12	5,9	1150	4.4						
13	8,4	700	3.7						
14	8,4	800	5.0						
15	13,4	950	5.2						
16	14,1	1000	4.7						
17	8,4	1050	4.9						
18	13,0	1150	5.1						
19	8,3	1000	5.3						
20	17,9	850	3.1						
21	18,1	900	2.6						
22	11,0	1000	2.9						
23	11,8	650	3.1						
24	19,1	950	3.5						
25	15,2	1000	4.0						
26	17,3	1250	4.1						
27	12,5	1300	3.6						
28	17,5	1100	3.8						
		VP			DA			D	
Animal	Angle (°)	Stim. Duration (ms)	Indentation	Angle (°)	Stim. Duration (ms)	Indentation	Angle (°)	Stim. Duration (ms)	Indentation
1	52,4	1250	5.7	35,4	800	5.4	44,1	900	6.1
2	59,4	1000	4.1	35,7	850	5.0	39,6	950	6.4
3	47,8	950	6.7	31,7	750	4.9	38,1	1050	5.7
4	44,4	1050	5.0	33,1	950	9.0	40,3	1200	4.3
5	49,4	900	7.1	22,4	900	7.8	45,4	1100	4.7
6	52,7	1100	5.6	20,4	1000	5.9	36,5	850	4.4
7	53,9	1150	5.7	17,0	1050	4.1	58,4	900	9.5
				29,1	850	8.7			
		DP							
	Angle (°)	Stim. Duration (ms)	Indentation						
1	43,9	1100	4.0						
2	42,3	1250	3.7						
3	39,4	1150	4.5						
4	45,1	1000	4.1						
5	34,5	950	3.9						
6	35,3	1050	5.0						
7	35,3	1200	4.8						
8	37,1	1150	5.2						
9	46,4	1000	4.4						
10	54,3	950	5.2						
